# Supplementary material for: A corrosion inhibiting layer to tackle the irreversible lithium loss in lithium metal batteries
Source: Nat Commun. 2023 Dec 13;14:8269. doi: 10.1038/s41467-023-44161-7 (PMC10719308; doi:10.1038/s41467-023-44161-7)
Supplement: Supplementary file 1 — Supplementary information [file 41467_2023_44161_MOESM1_ESM.pdf]

Supplementary information

A corrosion inhibiting layer to tackle the irreversible lithium loss in lithium metal batteries

Chengbin Jin[1*], Yiyu Huang[1], Lanhang Li[1], Guoying Wei[1], Hongyan Li[1], Qiyao Shang[2], Zhijin Ju[2], Gongxun Lu[2], Jiale Zheng[2], Ouwei Sheng[3*], Xinyong Tao[2*]

[1]College of Materials and Chemistry, China Jiliang University, Hangzhou 310018, China.

[2]College of Materials Science and Engineering, Zhejiang University of Technology, Hangzhou 310014, China.

[3]Institute of Advanced Magnetic Materials, College of Materials and Environmental Engineering, Hangzhou Dianzi University, Hangzhou 310012, China

*Corresponding author. Email: jincb@cjlu.edu.cn; owsheng@hdu.edu.cn; tao@zjut.edu.cn

Supplementary Notes 1–5

Supplementary Figs. 1–31

Supplementary Tables 1–3

Supplementary References

Supplementary Note 1. Features and prevention of different types of corrosion

Uniform corrosion. The most common type of corrosion caused by chemical or electrochemical attacks over the entire surface area of the metal[1].

Solutions: Corrosion allowance, alloy, and coatings.

Localized corrosion

Pitting corrosion. Localized dissolution of an oxide-covered metal in specific aggressive environments, resulting in small holes/pits[2].

Solutions: Cathodic protection, anodic protection, alloy, and selection of high-corrosion-resistance materials.

Crevice corrosion. Occurring in or directly adjacent to gaps or crevices on the surface of a metal[3].

Solutions: Crevices avoidance, cathodic protection, inhibitors, and coatings.

Filiform corrosion. Occurring underneath a coating and manifesting itself as threadlike fibers[4].

Solutions: Reducing the humidity, and multiple coats and proper alloy.

Deposition corrosion. A more noble metal depositing on the less noble metal to trigger pitting[5].

Solutions: Maintaining cleanliness, proper use of protective coatings/sealants, and addition of corrosion-resistant elements.

Galvanic corrosion/Bimetallic corrosion. Occurring between two dissimilar metals that are in electrical contact and exposed to electrolyte[6].

Solutions: Sacrificial material, selective coating, and insulating the galvanic couple.

Environmental cracking

Stress corrosion. Caused by a corrosive environment with residual mechanical stress on the material surfaces (*e.g.*, welding, heat treatments and cold deformations)[7].

Solutions: Stress relief (*e.g.*, annealing), surface treatments (*e.g.*, shot peening), and avoiding design of sharp corners, holes, *etc*. in materials.

Corrosion fatigue. Caused by a combination of cycling stress and corrosive environments[8].

Solutions: Alloys, coatings, inhibitors, reducing stress raisers, modifying the corrosive environment, and reducing residual stresses.

Hydrogen-induced cracking. Occurring in carbon or low-alloy steels when hydrogen atom diffuses into the materials and forms hydrogen molecules, internally pressurizing

the material and initiating cracking[9].

Solutions: Reducing regions of high micromechanical contrast, designing dense oxide surface layers, and trapping hydrogen at proper internal interfaces and defects.

Flow-assisted corrosion

Erosion corrosion. Triggered by the combined mechanical stress action from the fluid motion, and the corrosive chemical actions in the fluids[10].

Solutions: Using coatings and other surface treatment, and cathodic protection.

Cavitation corrosion. Caused by the formation and collapse of gas bubbles on the surface of the material, inducing initial cavitation[11].

Solutions: Ceramic/epoxy/polyurethane coatings, weld overlayers, and plasma/thermal spray treatments.

Impingement corrosion. Localized erosion-corrosion caused by turbulence or impinging flow, damaging the protective oxide film[12].

Solutions: Corrosion-resistant coatings.

Intergranular corrosion. A chemical or electrochemical attack on or adjacent to the grain boundaries of a metal[13].

Solutions: Conducting proper annealing and quenching treatments, and regulating the alloy compositions.

Selective leaching corrosion/De-alloying. Preferential removal of one constituent of an alloy to leave behind an altered residual structure (*e.g.*, de-zincification of brass)[14].

Solutions: Using cladding process, adding proper element, and cathodic protection.

Fretting corrosion. Caused by small cyclic movements between two materials associated with corrosive attack from the environment[15].

Solutions: Reducing the relative motion, increasing the interfacial stability, and using anti-fretting coatings, shot peening, and lubrication.

High-temperature corrosion. A chemical attack from gases, solid or molten salts, or molten metals at high temperatures[16].

Solutions: Overlay and thermal barrier coatings.

Biological corrosion. Caused by biological organisms or microorganisms[17].

Solutions: Coatings, cathodic protection, biocides, and cleaning procedures.

Supplementary Note 2. Characterization techniques in corrosion science

Typical characterization techniques in materials science on microstructure and chemistry examination can be successfully translated into the study of corrosion science. Additionally, electrochemical measurements are also important techniques for quantitative corrosion researches. The techniques for corrosion research have been summarized in Supplementary Table 1. Last but not the least, computational and artificial intelligence are promising in the exploration and investigation of corrosion science.

Supplementary Note 3. The similarities and differences between typical corrosion and Li corrosion

Similarity: 1) Li like typical structural metals can undergo various kinds of corrosion due to the interaction between metals and reactive environments, including pitting, stress corrosion, intergranular corrosion, and galvanic corrosion in contact with a noble metal[18,19]; 2) Corrosion of Li and structural metals will trigger the changes/deterioration of their structures, chemistries, and functions; 3) Corrosion leads to the formation of passive layers on both metal surfaces, which is comprised of corrosion products; 4) The corrosion of Li and structural metals both relates to the breakage/failure of passive layers. For example, stainless steels are susceptible to pitting corrosion due to the localized dissolution of an oxide-covered metal in aggressive environments[20]. In terms of the passive layer of Li (*i.e.*, SEI), it suffers mechanical and chemical instability. Due to the cracks and dissolution of SEI, Li exposed to the electrolyte will be continuously corroded; 5) The corrosion prevention strategies in typical corrosion science can be successfully translated into battery systems, including the coating, doping, and regulation of the alloy compositions; 6) The characterization techniques of corrosion research can also be adopted in battery systems, such as the electron backscatter diffraction.

Difference: 1) Overpotentials are less likely to occur during Li corrosion due to the extremely low redox potential of Li and sufficiently fast Li^+ diffusion through the SEI[21]. Conversely, corrosion of structural metals usually involves overpotentials against metal plating, spatial segregation of cathodic and anodic processes, and kinetics considerations[22]; 2) The compositions and functions of passive layers on Li and

structural metals are different. The passive layer of structural metals formed in atmospheric environments or aqueous solutions mainly consists of inorganic oxide or hydroxide, some of which can suppress the proceeding of corrosion (*e.g.*, Al_2O_3 on Al), while others cannot inhibit the corrosion (*e.g.*, rust on steel). In contrast, the passive layer on Li (*i.e.*, SEI) usually comprises of organic and inorganic Li salts, which is supposed to inhibit the continuous corrosion reactions, meanwhile enabling the reversible operation of batteries (*i.e.*, charging/discharging, or plating/stripping); 3) Li with high redox power, serious volumetric effect, and instable SEI will be repeatedly exposed to the electrolyte, making the prevention of Li corrosion much more challenging than that of structural metal; 4) Since Li is sensitive to heat, electron, and air, expeditions on Li corrosion are faced with greater difficulty than that of the structural metal corrosion. The investigation of Li corrosion depends more tightly on advanced characterization techniques.

Supplementary Note 4. Issues and topics in corrosion science

The issues and topics in corrosion science presently include[23,24]: 1) Enhancing the strength and corrosion resistance of a material concurrently. The high strength of materials mainly comes from complex microstructures, chemical compositions and internal stress fields, which make such materials susceptible to corrosion; 2) Increasing the sustainability/recyclability of alloys and maintaining the corrosion resistance; 3) Designing advanced corrosion inhibitors, coatings, and cathodic protection techniques; 4) Developing ab initio-based computational and artificial intelligence in corrosion science; 5) Designing analytical and in-operando methods for high-resolution and quantitative corrosion probing, especially for hydrogen induced corrosion; 6) Developing hydrogen-embrittlement-resistant materials for the use of hydrogen as an energy carrier (*e.g.*, doped medium- and high-entropy alloy variants); 7) Corrosion in the oil and gas industry, nuclear facilities, and battery. Notably, the corrosion in battery systems is thought to be an important topic for both corrosion science and battery research.

Supplementary Note 5. The significance of corrosion science in battery systems

Battery materials including electrodes, current collector, and solid-state electrolytes are susceptible to corrosion, which largely deteriorate the battery performances and should be seriously considered. Notably, corrosion in batteries exhibits the typical features of corrosion, while it also distinguishes from the corrosion of structural metals, especially for Li metal negative electrode of ultra-active nature and complex SEI. The synergistic investigations of corrosion science and corrosion prevention in Li metal batteries will yield interdisciplinary benefits to both fields: 1) New recognition on battery failure mechanism (hydrogen, stress, *etc*. related corrosion); 2) Guiding the design and strategies for batteries with longevity; 3) Inspiring the corrosion prevention in other fields; 4) Promoting the development of advanced characterization techniques; 5) Extending and establishing corrosion science in battery systems.

Supplementary figures

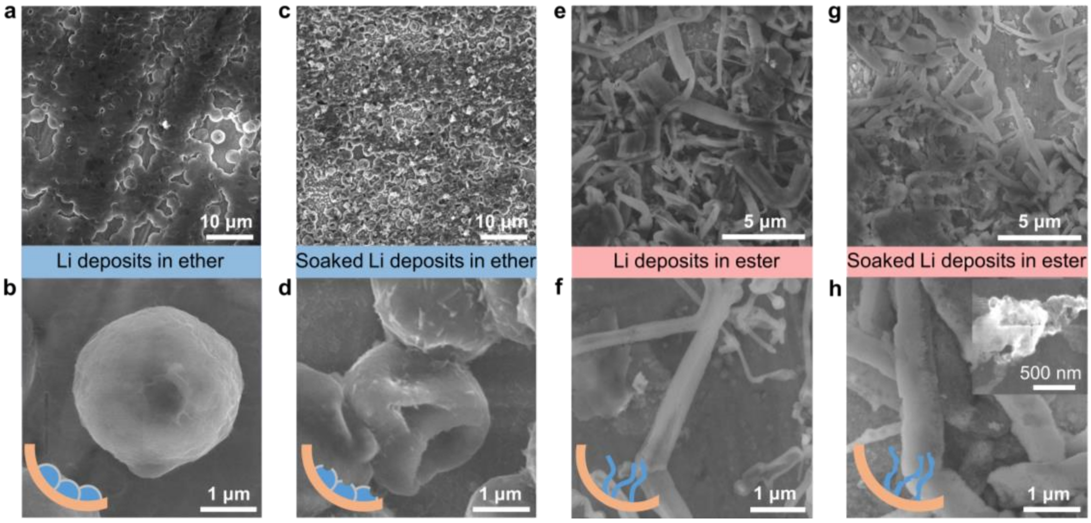

Supplementary Fig. 1 | SEM images of Li deposits before and after soaking in the ether and ester electrolytes. a, b SEM images of Li deposits formed in 1.0 M LiTFSI in DOL/DME with 1.0 wt% $LiNO_3$. **c, d** SEM images of Li deposits formed in 1.0 M LiTFSI in DOL/DME with 1.0 wt% $LiNO_3$ after soaking in the electrolyte. **e, f** SEM images of Li deposits formed in 1.0 M $LiPF_6$ in EC/EMC/DEC with 1.0 wt% FEC. **g, h** SEM images of Li deposits formed in 1.0 M $LiPF_6$ in EC/EMC/DEC with 1.0 wt% FEC after soaking in the electrolyte.

At a low depositing capacity like 0.5 mAh cm^{-2}, the Cu current collector will not be totally covered by Li, making the proceeding of both chemical and electrochemical corrosion of Li. The corroded morphology of Li deposits can be clearly seen in the SEM images.

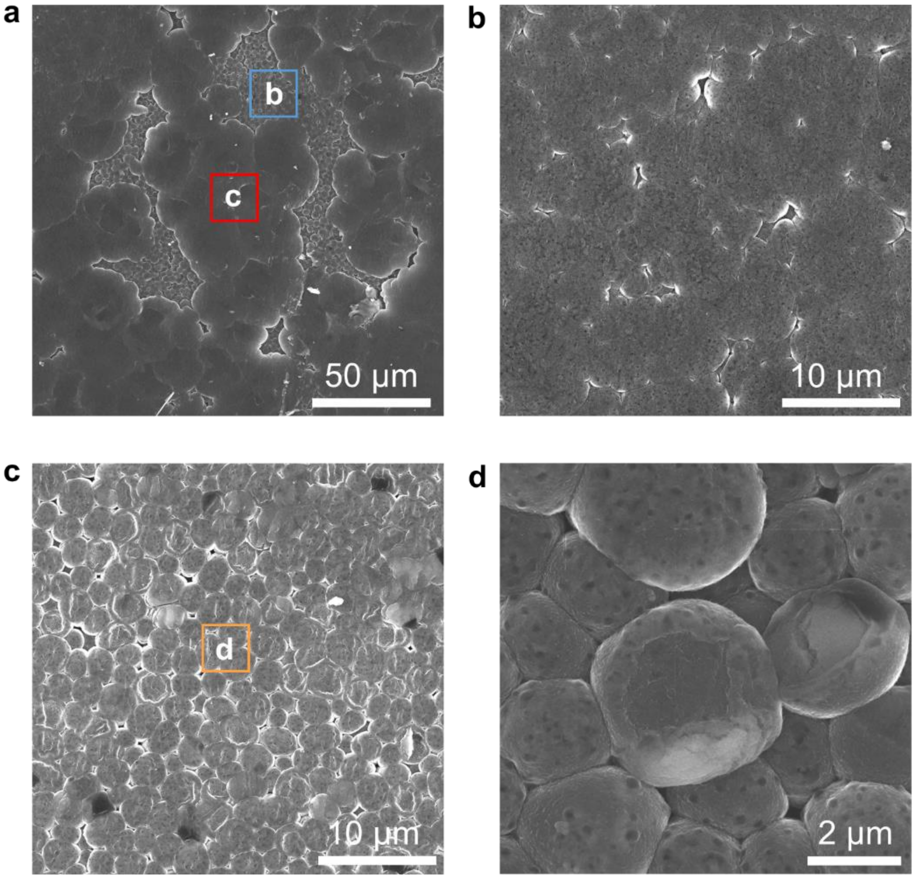

Supplementary Fig. 2 | Morphologies of Li deposits after soaking in 1.0 M LiTFSI in DOL/DME with 1.0 wt% $LiNO_3$. a–d SEM images of Li deposits formed at 1.0 mA cm^{-2} with a capacity of 3.0 mAh cm^{-2}.

Even at a large Li deposition capacity where Cu was totally covered by Li, Li corrosion still can be observed in the Li deposits, which can be ascribed to the corrosion patterns like pitting, intergranular corrosion and/or chemical corrosion. Additionally, during the subsequent Li stripping, fresh Cu surface covered with Li deposits will be progressively exposed, again initiating the galvanic corrosion[25]. Besides, Li metal negative electrode will become loose and porous after repeated cycling, where the dynamic galvanic corrosion potentially occurs with the penetration of electrolyte.

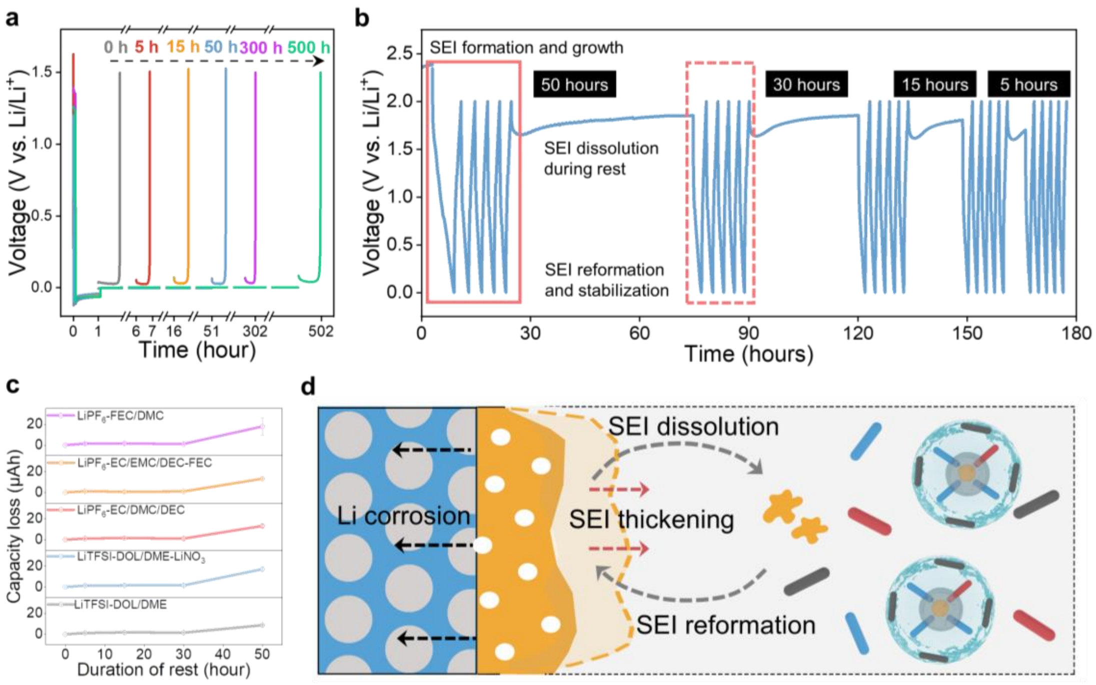

Supplementary Fig. 3 | Electrochemical protocols for detecting Li corrosion and SEI dissolution. a Voltage profiles of Li||Cu cells with different resting time for detecting Li corrosion. The cell was cycled at 1.0 mA cm^{-2} and 25 °C with a Li cycling capacity of 1.0 mAh cm^{-2}. **b** Voltage profiles of Li | Cu cells in the range from 0.05 V to 2.0 V with different resting time for detecting SEI dissolution. The cell was cycled at 6.0 μA and 25 °C, and the electrolyte used was 1.0 M LiTFSI in DOL/DME with 1.0 wt% $LiNO_3$. **c** SEI dissolution related capacity loss of active Li sources in various electrolytes as a function of resting time. The electrolytes include 1.0 M LiTFSI in DOL/DME (v/v=1:1), LiTFSI in DOL/DME with 1.0 wt% $LiNO_3$ (v/v=1:1), $LiPF_6$ in EC/DMC/DEC (v/v/v=1:1:1), $LiPF_6$ in EC/EMC/DEC with 1.0 wt% FEC (v/v/v=1:1:1), and $LiPF_6$ in FEC/DMC (v/v=1:4). The error bar corresponded to standard deviation from the measurements with three identical cells. **d** A schematic illustration of the SEI progression, and its correlation with the net growth of SEI and continuous Li corrosion.

The slight increase was observed in the cell voltage during the pause, which suggested that the SEI was not sufficiently stable and dissolved in the electrolyte. Consequently, extra reductive capacity is required to supplement the loss of SEI. Such changes of reductive capacity can be applied to quantitatively describe the behavior of SEI dissolution.

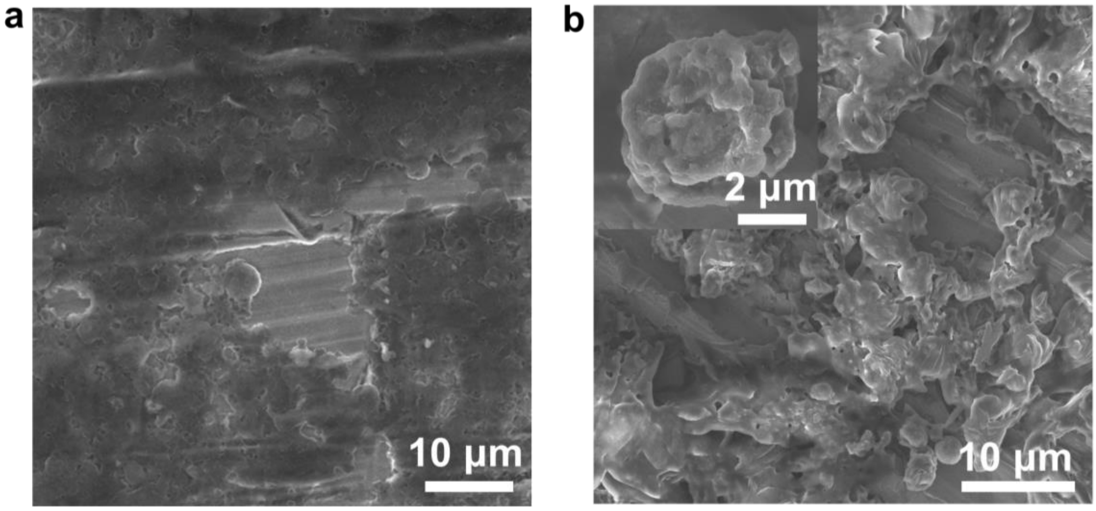

Supplementary Fig. 4 | Morphologies of SEI before and after soaking in the electrolytes. a, b SEM images of SEI formed in 1.0 M LiTFSI in DOL/DME with 1.0 wt% $LiNO_3$ before and after soaking. The SEI was produced after one Li plating/stripping at 1.0 mA cm^{-2} with a cycling capacity of 0.5 mAh cm^{-2}.

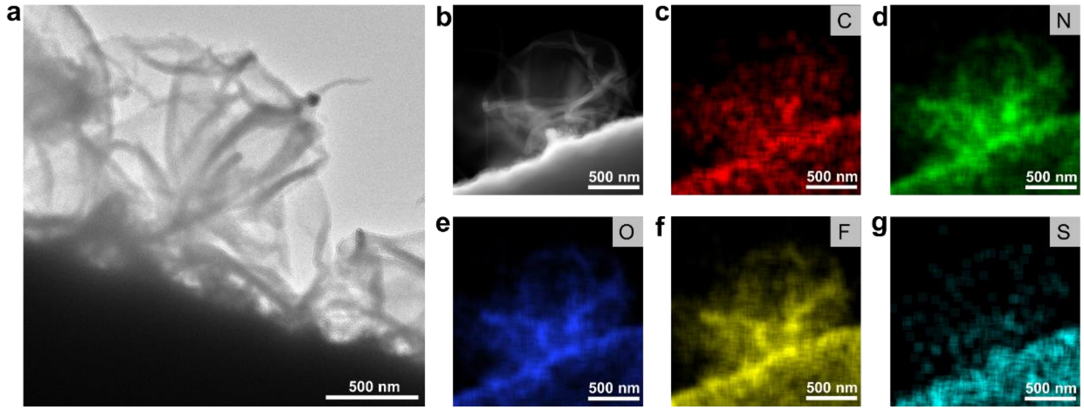

Supplementary Fig. 5 | Cryo-TEM images of SEI residual on bare Cu grid. a–g Cryo-TEM image, HAADF image, and elemental mappings of SEI residual on bare Cu grid. The SEI residual was formed in 1.0 M LiTFSI in DOL/DME with 1.0 wt% $LiNO_3$ after one Li plating/stripping process, which was then quickly washed with DOL to dissolve the residual LiTFSI salt.

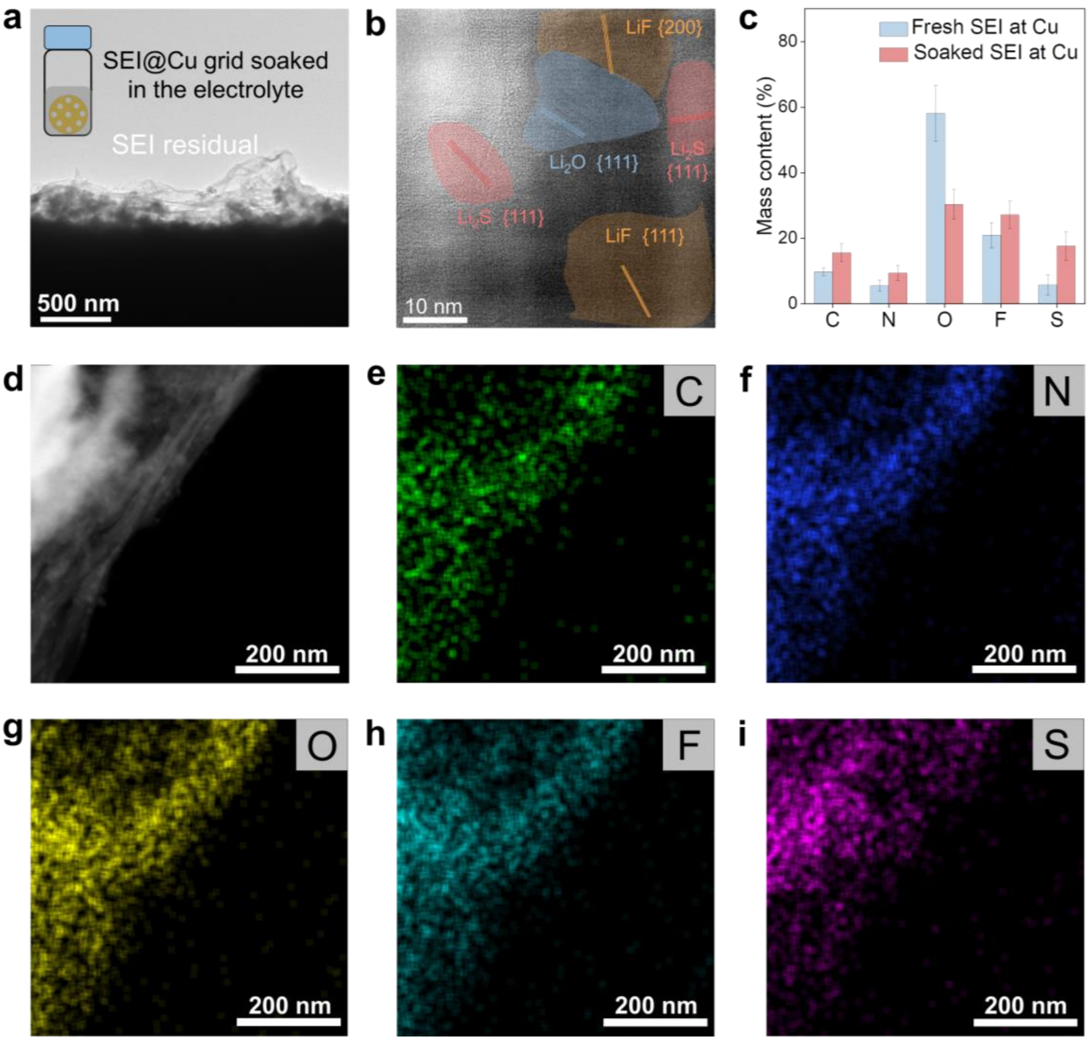

Supplementary Fig. 6 | Cryo-TEM images of SEI residual on bare Cu grid after soaking. a, b Cryo-TEM image and HRTEM image of SEI residual after soaking in the electrolyte. **c** The mass contents of various elementals in SEI before and after soaking in the electrolyte. The error bar corresponded to standard deviation from the measurements with three identical samples. **d–i** HAADF image and elemental mappings of SEI residual after soaking in the electrolyte. The SEI residual was formed in 1.0 M LiTFSI in DOL/DME with 1.0 wt% $LiNO_3$ after one Li plating/stripping process, which was subsequently soaked in the same electrolyte.

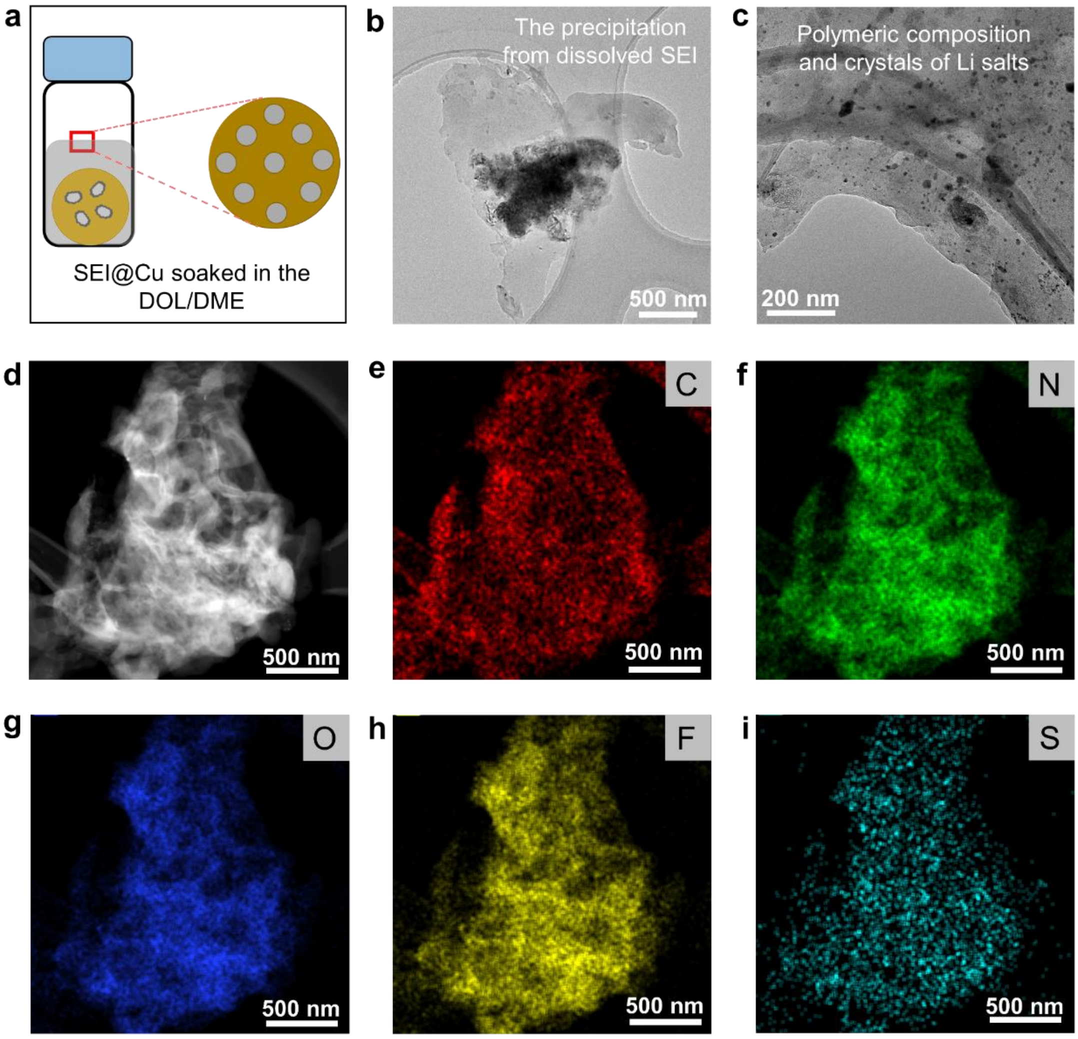

Supplementary Fig. 7 | Cryo-TEM images of precipitation from dissolved SEI. a Schematic illustration showing the preparation of sample by drying the DOL/DME solution containing dissolved SEI. **b, c** TEM and HRTEM images of precipitation after drying the DOL/DME solution containing dissolved SEI. **d–i** HAADF image and elemental mappings of the precipitation from dissolved SEI. The precipitation from dissolved SEI was obtained by dissolving SEI in the mixed solvent of DOL/DME, after which the transparent solution containing dissolved SEI was transferred onto the Cu grid and dried.

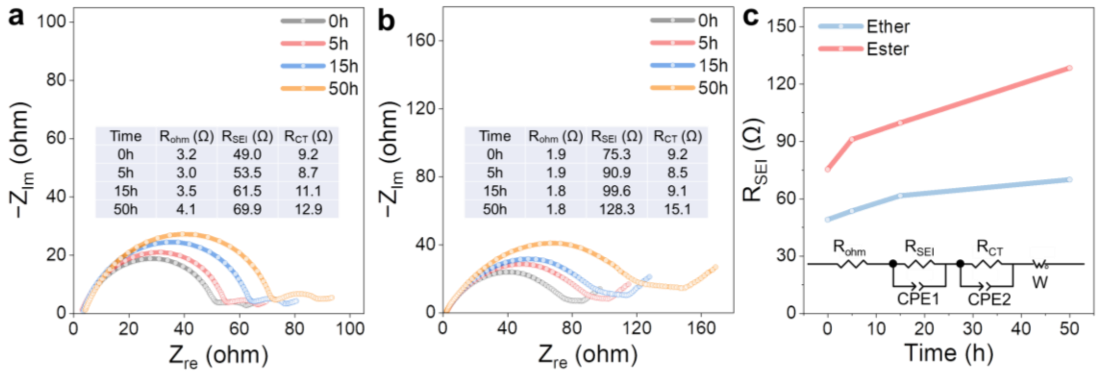

Supplementary Fig. 8 | EIS results of Li||Li@Cu. a EIS curves of Li||Li@Cu after different resting time in 1.0 M LiTFSI in DOL/DME with 1.0 wt% $LiNO_3$. **b** EIS curves of Li||Li@Cu after different resting time in 1.0 M $LiPF_6$ in EC/EMC/DEC with 1.0 wt% FEC. Li of 0.5 mAh cm^{-2} was plated onto the Cu in Li||Cu cell at 1.0 mA cm^{-2}. The fitted results of impedance were presented in the insets. **c** R_{SEI} of cells with ether and ester electrolytes after different resting time.

With the increase of resting time, the impedance of SEI gradually enlarges, indicating the dynamic evolutions of SEI. The repeated breakage/dissolution and repair/reformation of SEI will result in the continuous loss of active Li source and the net growth of SEI.

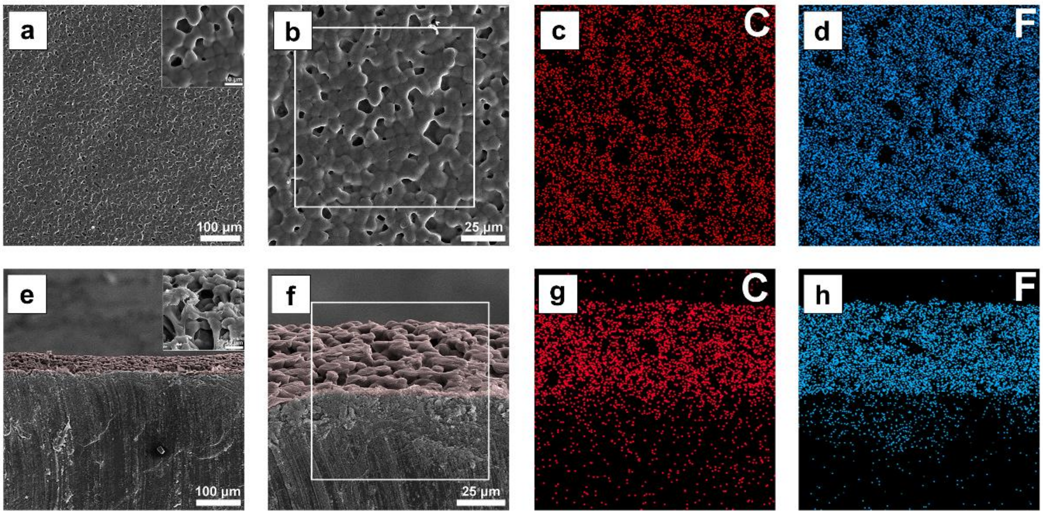

Supplementary Fig. 9 | SEM images and elemental mappings of PVDF-Li. a Top-view SEM image of PVDF-Li. **b–d** SEM image and corresponding elemental mappings of PVDF-Li. **e** Cross-section SEM image of PVDF-Li. **f–h** SEM image of top layer at PVDF-Li from cross section and corresponding elemental mappings.

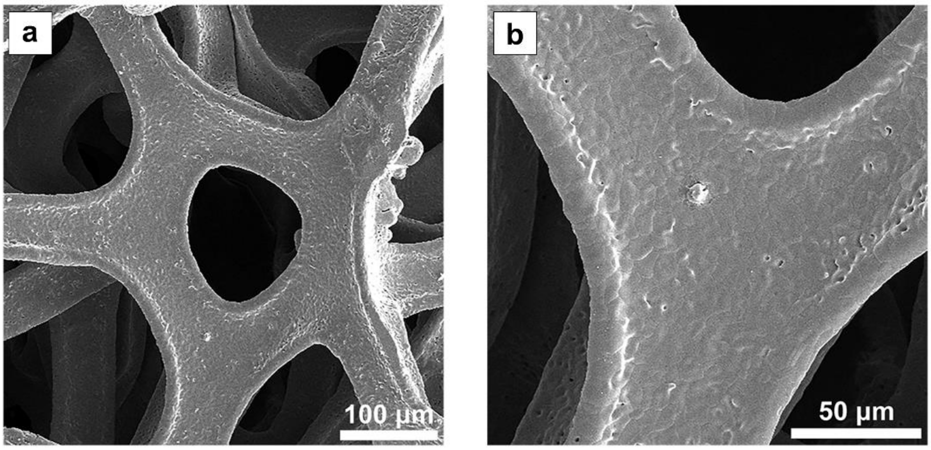

Supplementary Fig. 10 | Morphology of Cu foam. a, b SEM images of bare Cu foam.

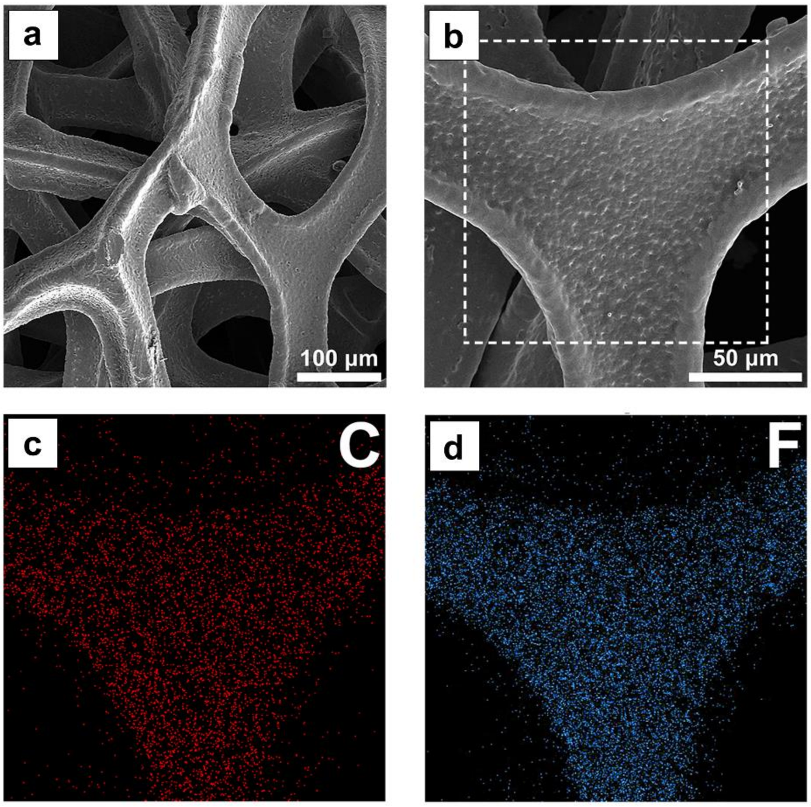

Supplementary Fig. 11 | Morphology of PVDF-Cu. a, b SEM images of PVDF-Cu. **c, d** The corresponding elemental mappings for C and F.

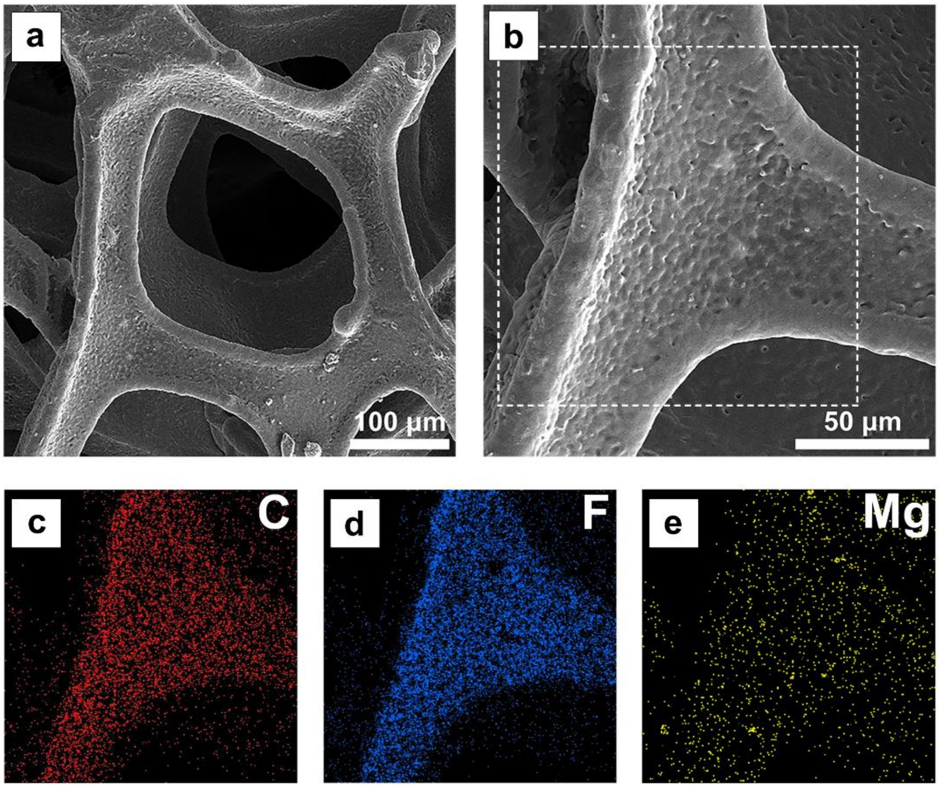

Supplementary Fig. 12 | Morphology of MgF_2@PVDF-Cu. a, b SEM images of MgF_2@PVDF-Cu. **c–e** The corresponding elemental mappings for C, F, and Mg.

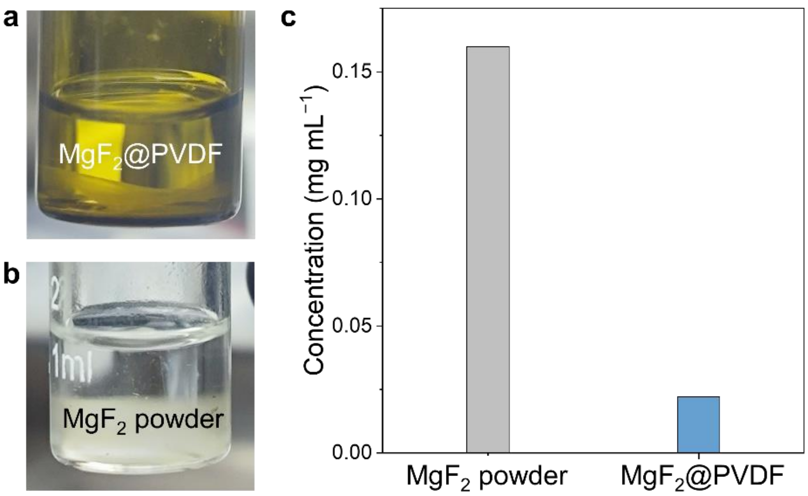

Supplementary Fig. 13 | Solubility of MgF_2. a, b Digital photos of MgF_2@PVDF layer and MgF_2 powder soaked in the DOL/DME. **c** The concentration of Mg^{2+} in the solution containing MgF_2@PVDF layer or MgF_2 powder.

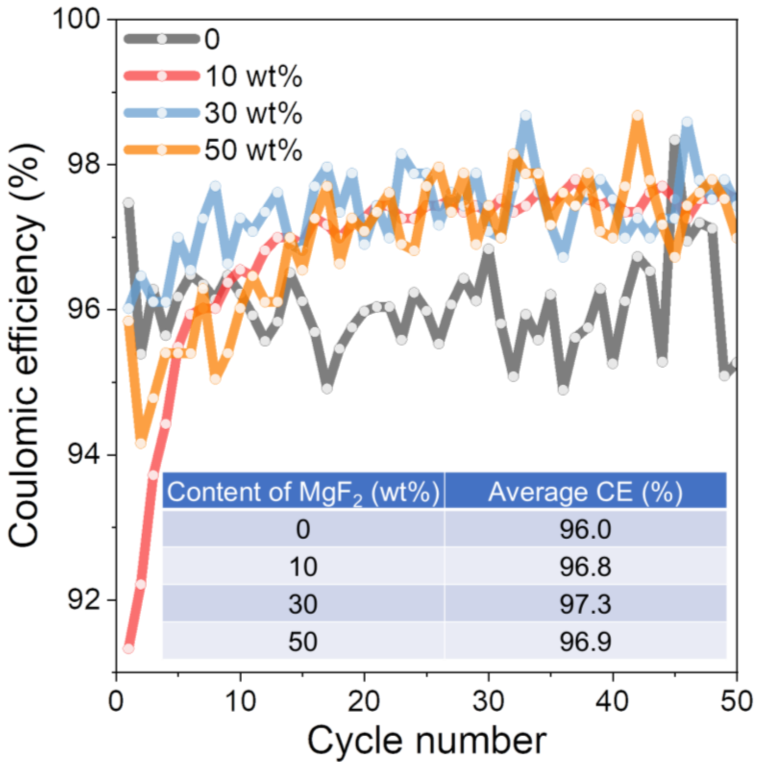

Supplementary Fig. 14 | CE results of Li||Cu cells with artificial passivation layers of different contents of MgF_2. The cells were tested at 1.0 mA cm^{-2} with a Li cycling capacity of 1.0 mAh cm^{-2}.

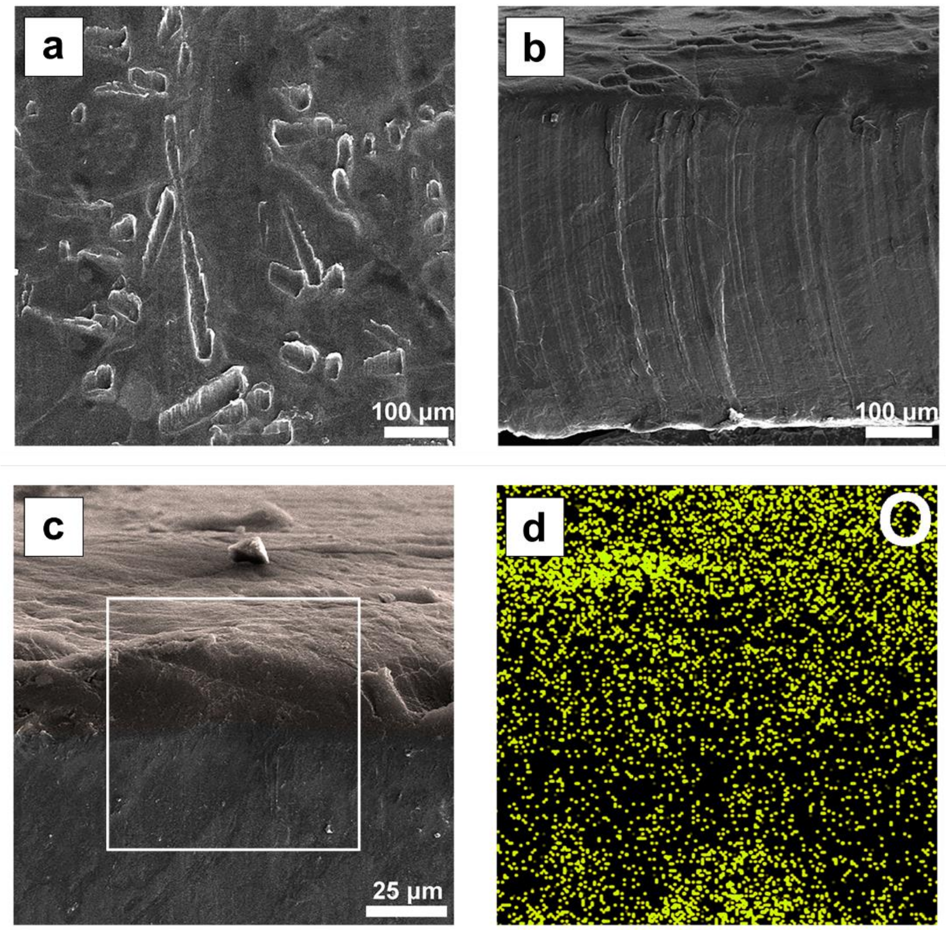

Supplementary Fig. 15 | SEM images and elemental mapping of pristine Li foil. a Top-view SEM image of Li. **b** Cross-section SEM image of Li. **c, d** SEM image of the top layer of Li foil from cross section, and the elemental mapping for O.

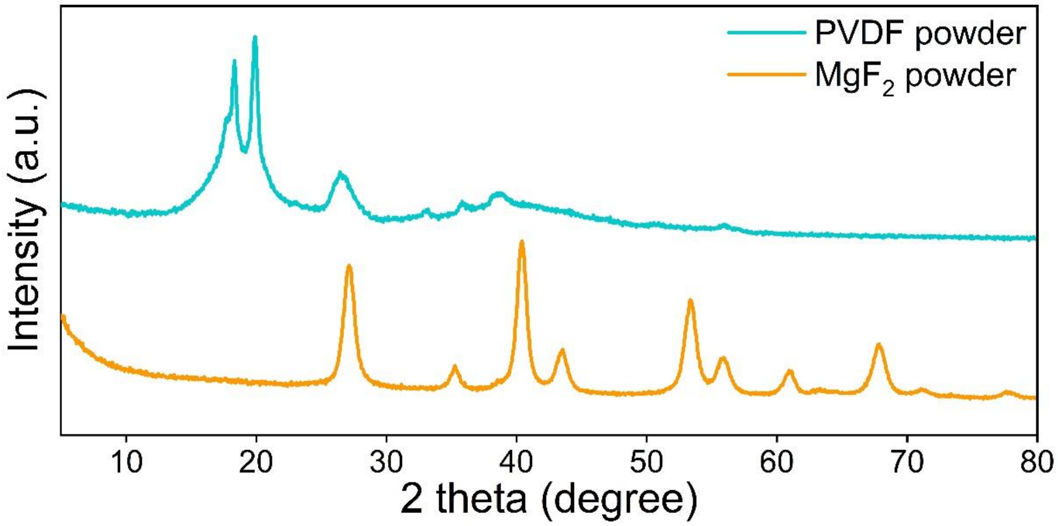

Supplementary Fig. 16 | XRD patterns of PVDF and MgF_2 powder.

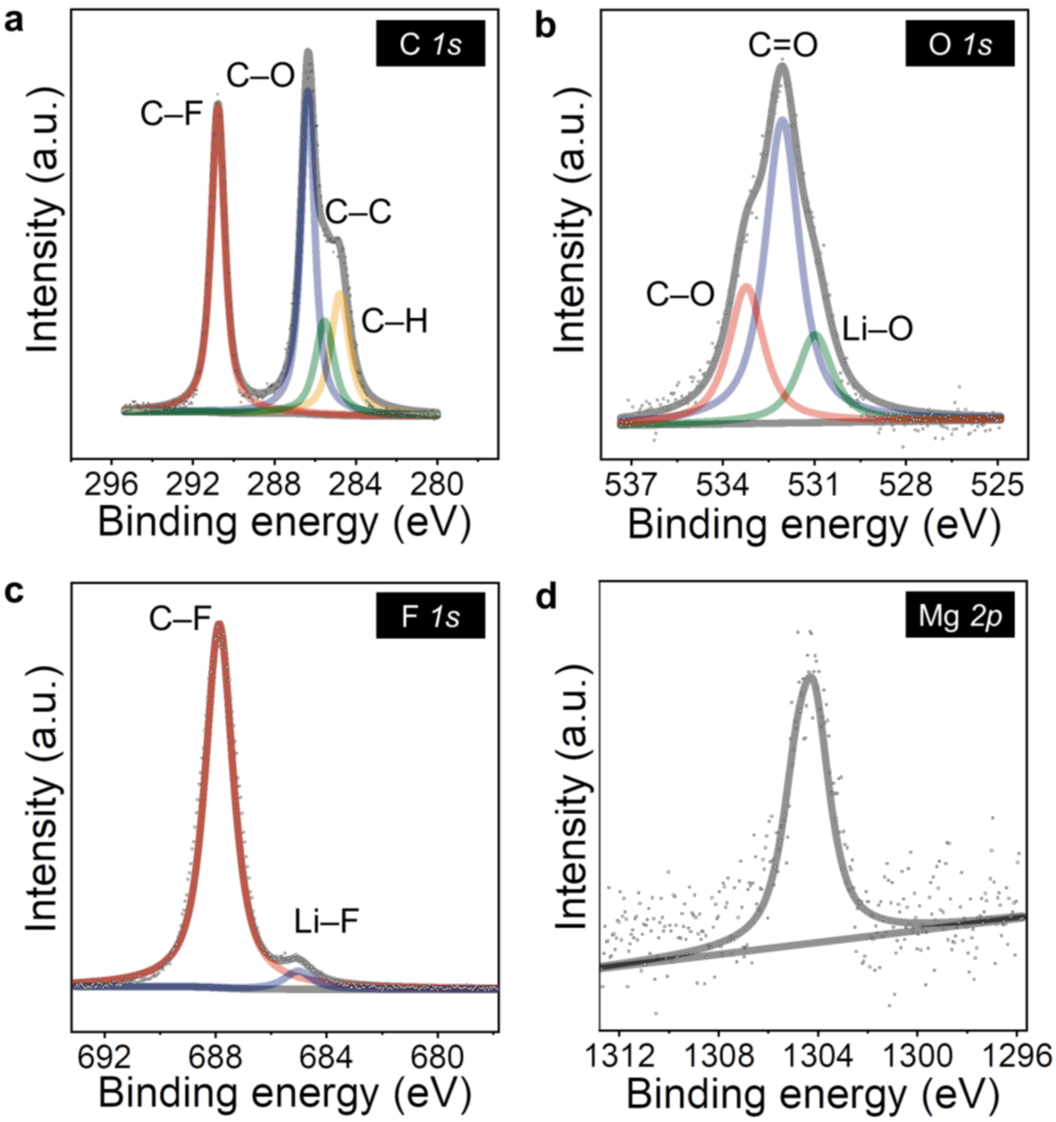

Supplementary Fig. 17 | XPS results of MgF_2@PVDF passivated Li. a–d XPS results of C *1s*, O *1s*, F *1s*, and Mg *2p* of MgF_2@PVDF passivated Li, respectively.

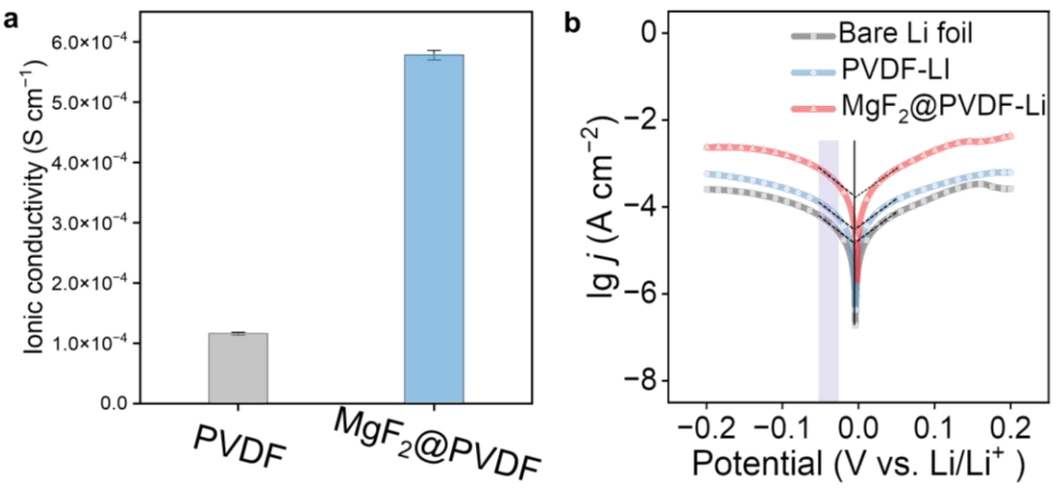

Supplementary Fig. 18 | Ionic conductivity and exchange current densities. a Ionic conductivity of PVDF and MgF_2@PVDF layer absorbing 1.0 M LiTFSI in DOL/DME with 1.0 wt% $LiNO_3$. The error bar corresponded to standard deviation from three independent measurements. **b** Tafel plots obtained from Li||Li symmetric cells with bare Li, PVDF-Li, and MgF_2@PVDF-Li.

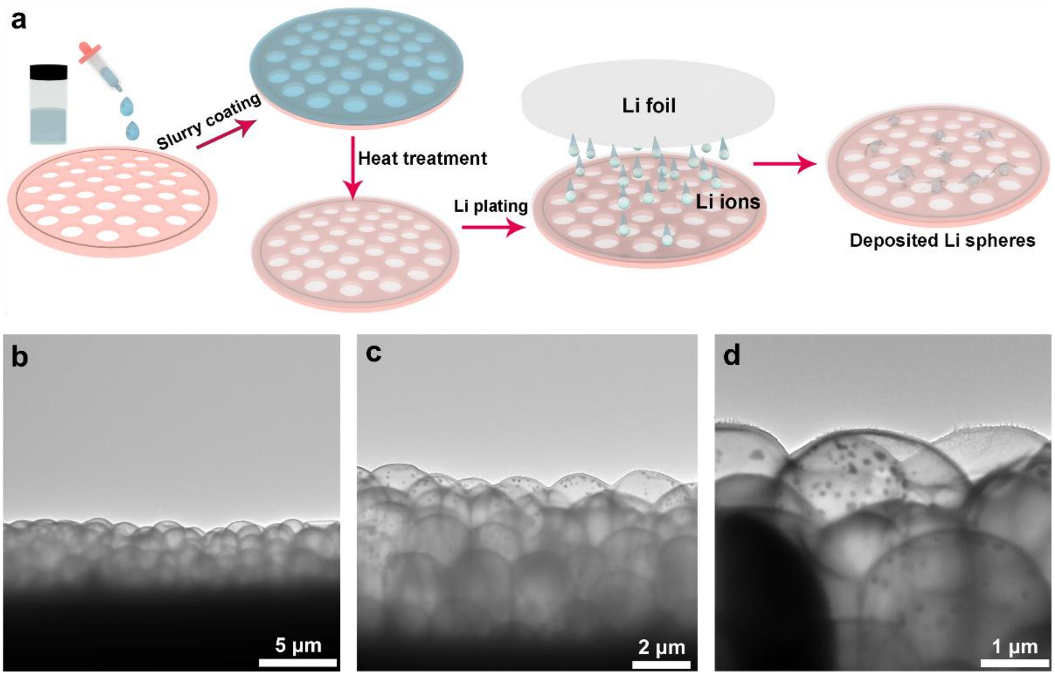

Supplementary Fig. 19 | Preparation and characterization of MF_x@PVDF. a Preparation of Cu grid with a MF_x@PVDF layer and Li deposits on MF_x@PVDF protected Cu grid. **b–d** Cryo-TEM images of Li deposits on Cu grid with an artificial passivation layer.

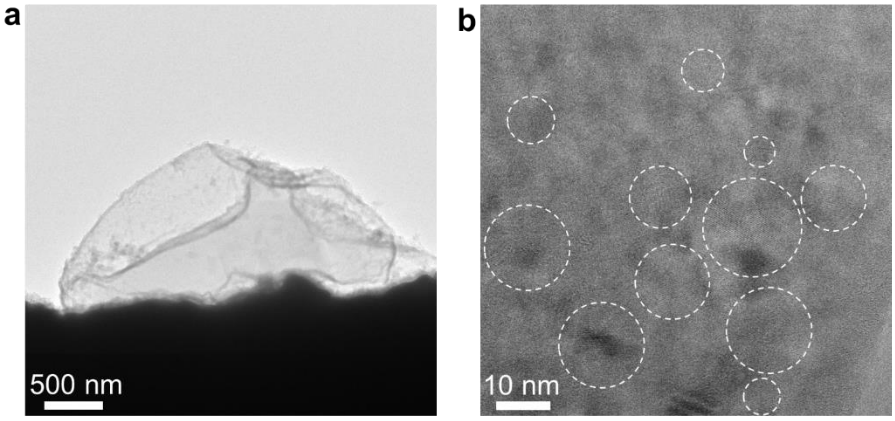

Supplementary Fig. 20 | Cryo-TEM images of corroded Li deposits on bare Cu grid. a Cryo-TEM image of corroded Li. **b** HRTEM images of corroded Li with abundant crystals like Li_2O. The Li deposits were formed in 1.0 M LiTFSI in DOL/DME with 1.0 wt% $LiNO_3$.

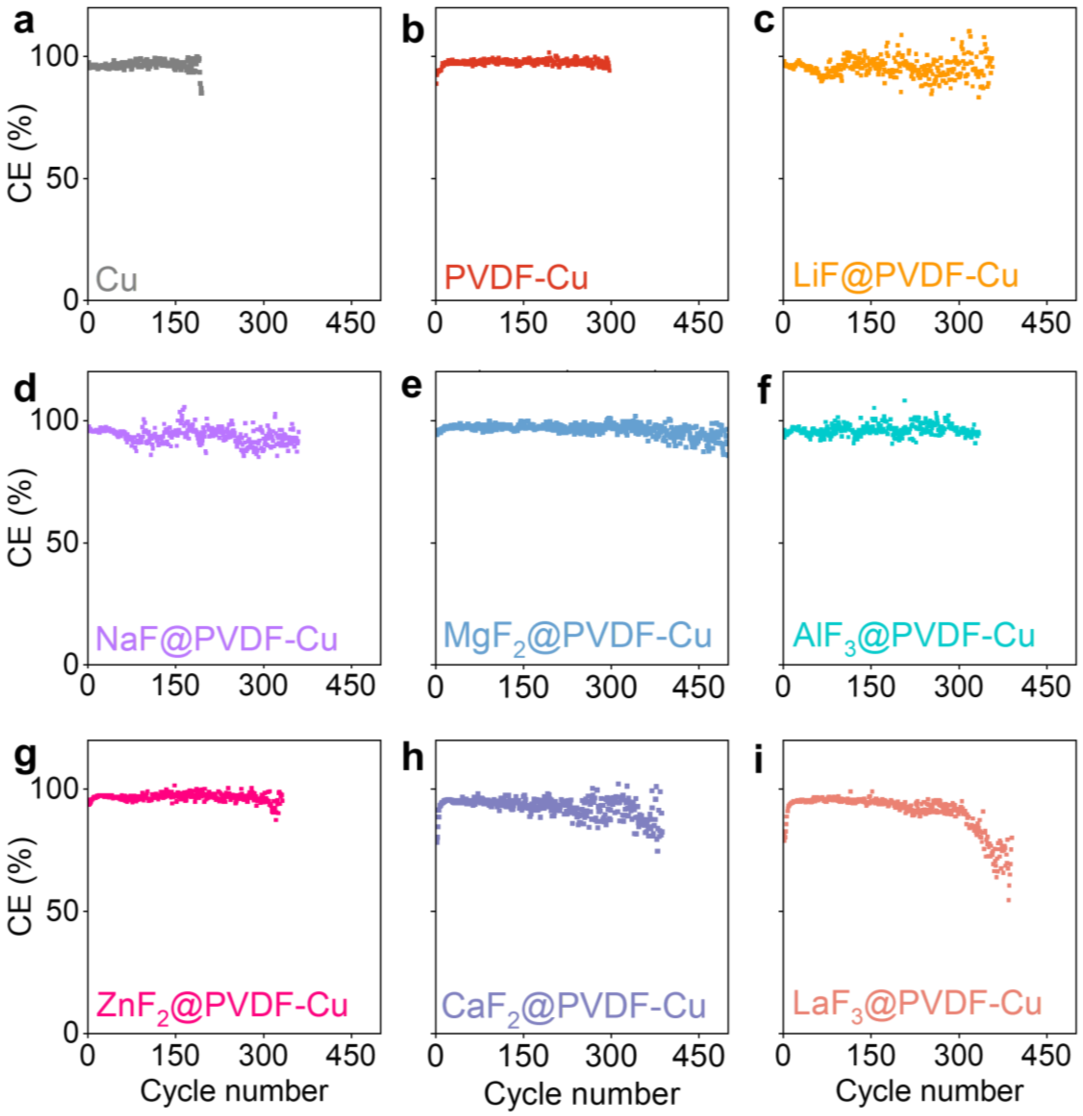

Supplementary Fig. 21 | CE results of various electrodes at 1.0 mA cm^{-2} with a Li cycling capacity of 1.0 mAh cm^{-2}. a Bare Cu. **b** PVDF-Cu. **c** LiF@PVDF-Cu. **d** NaF@PVDF-Cu. **e** MgF_2@PVDF-Cu. **f** AlF_3@PVDF-Cu. **g** ZnF_2@PVDF-Cu. **h** CaF_2@PVDF-Cu. **i** LaF_3@PVDF-Cu.

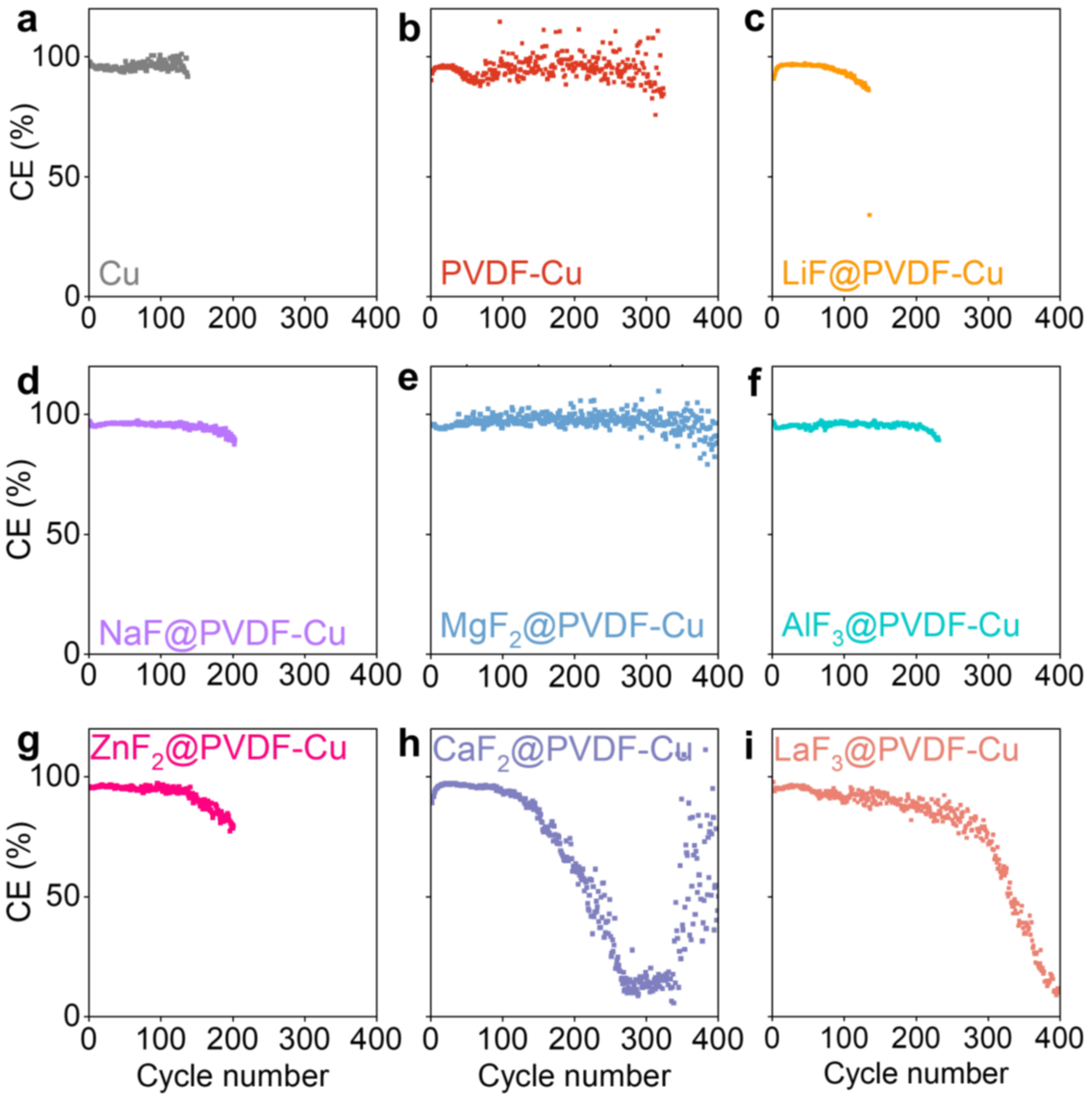

Supplementary Fig. 22 | CE results of various electrodes at 3.0 mA cm^{-2} with a Li cycling capacity of 1.0 mAh cm^{-2}. **a** Bare Cu. **b** PVDF-Cu. **c** LiF@PVDF-Cu. **d** NaF@PVDF-Cu. **e** MgF_2@PVDF-Cu. **f** AlF_3@PVDF-Cu. **g** ZnF_2@PVDF-Cu. **h** CaF_2@PVDF-Cu. **i** LaF_3@PVDF-Cu.

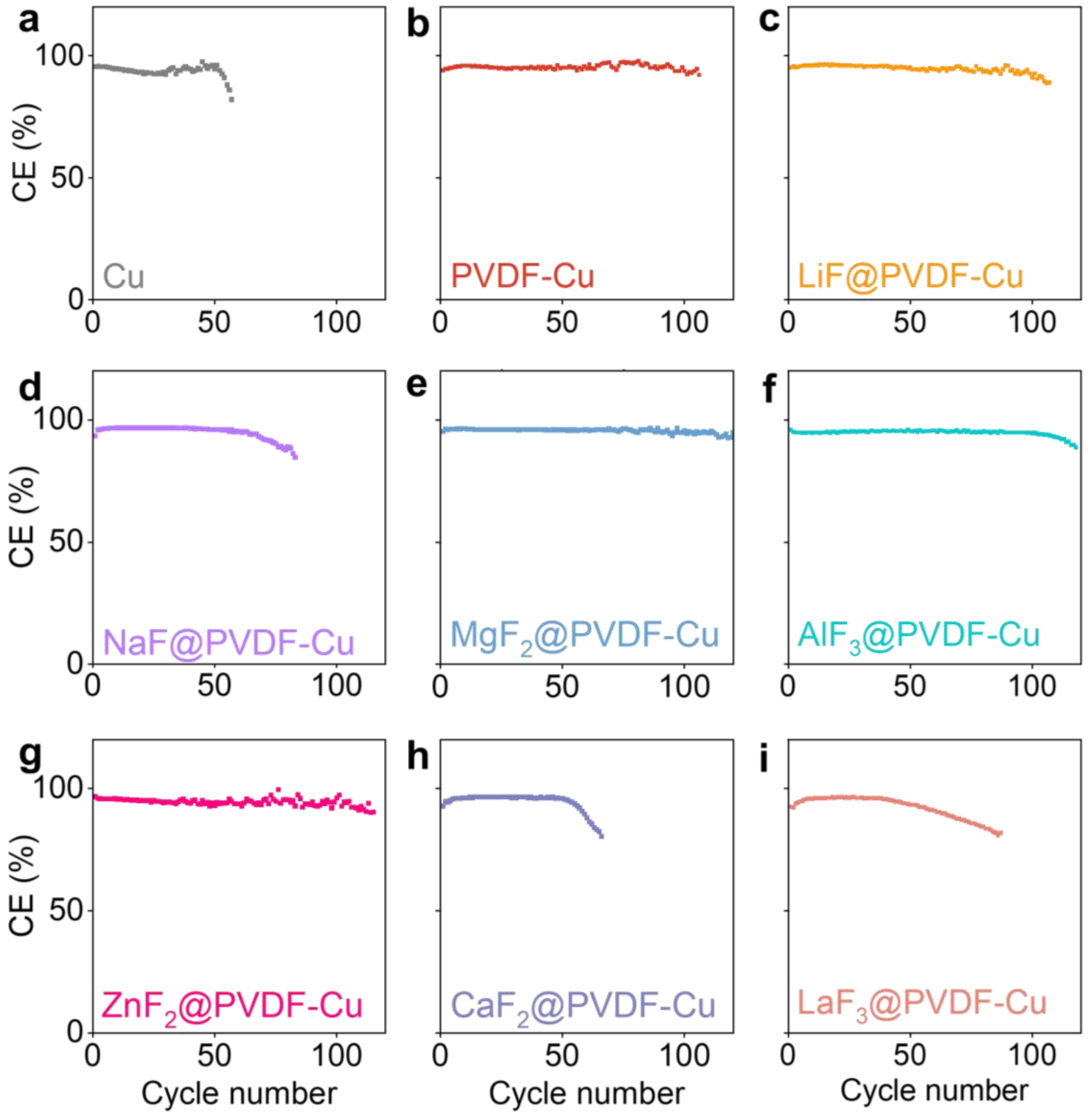

Supplementary Fig. 23 | CE results of various electrodes at 5.0 mA cm^{-2} with a Li cycling capacity of 1.0 mAh cm^{-2}. **a** Bare Cu. **b** PVDF-Cu. **c** LiF@PVDF-Cu. **d** NaF@PVDF-Cu. **e** MgF_2@PVDF-Cu. **f** AlF_3@PVDF-Cu. **g** ZnF_2@PVDF-Cu. **h** CaF_2@PVDF-Cu. **i** LaF_3@PVDF-Cu.

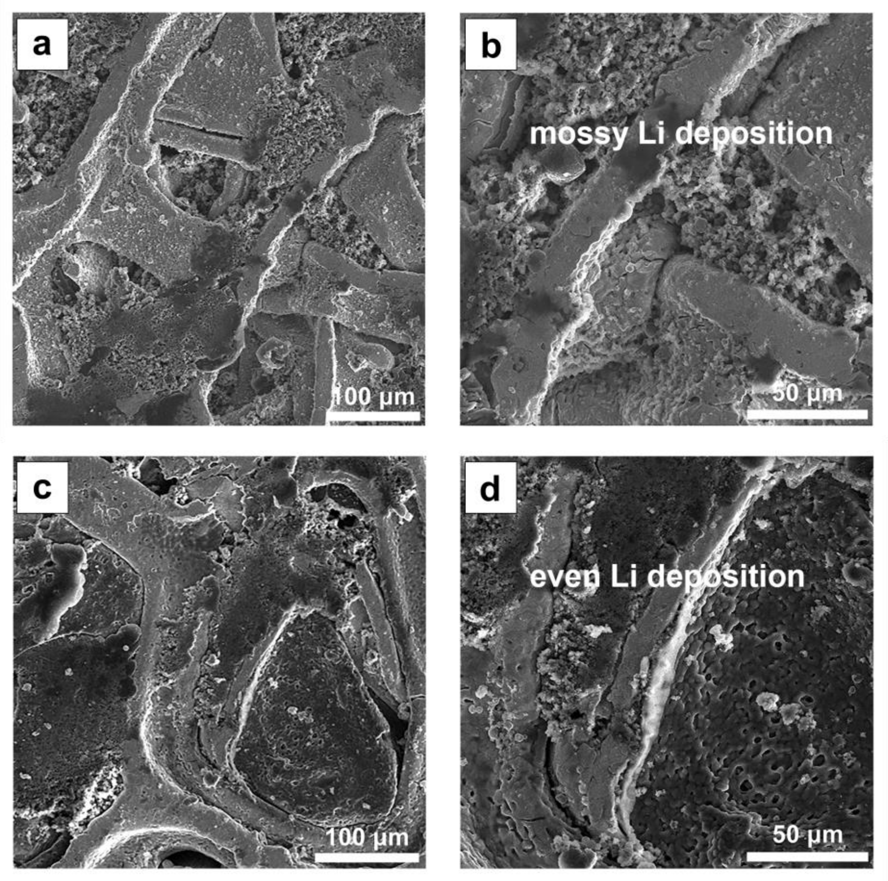

Supplementary Fig. 24 | Morphology of Cu foams after Li deposition. a, b SEM images of Cu foam after Li deposition. **c, d** SEM images of MgF_2@PVDF-Cu after Li deposition. The electrolyte used was 1.0 M LiTFSI in DOL/DME with 1.0 wt% $LiNO_3$.

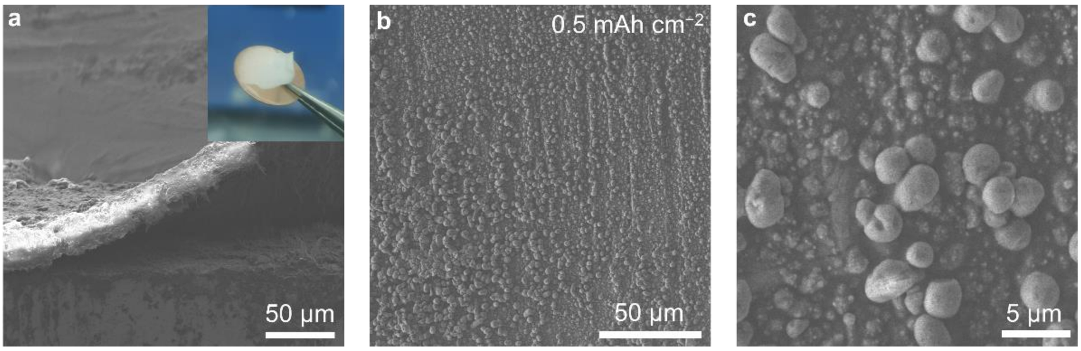

Supplementary Fig. 25 | Li deposits underneath the MgF_2@PVDF. a Cross-sectional SEM image of Li deposits on MgF_2@PVDF layer coated Cu foil. Inset is the digital photo of MgF_2@PVDF layer coated Cu foil, and the artificial passivation layer was peeled off to show the Li deposits. **b, c** Top-view SEM images of Li deposits underneath the MgF_2@PVDF layer. The electrolyte used was 1.0 M LiTFSI in DOL/DME with 1.0 wt% $LiNO_3$.

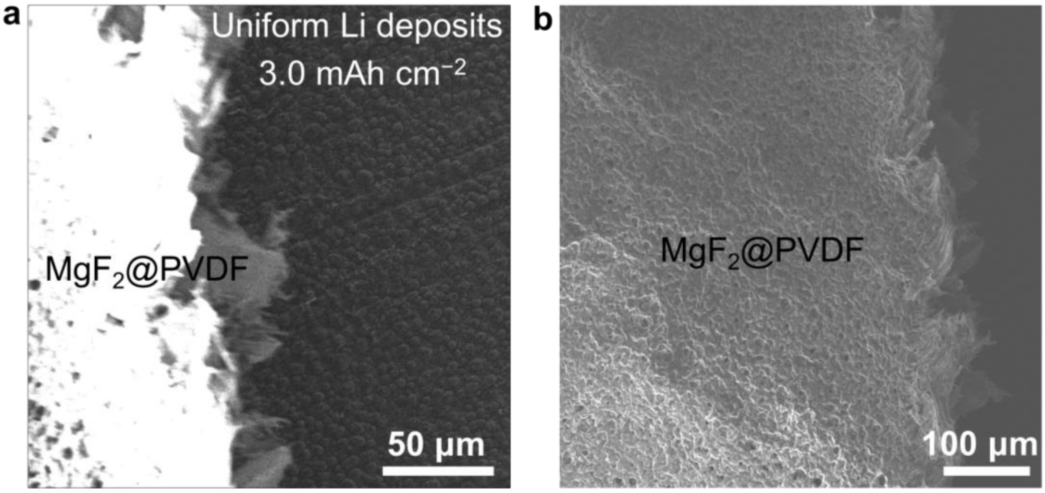

Supplementary Fig. 26 | Li deposits underneath the MgF_2@PVDF. a, b SEM image of Li deposits underneath the MgF_2@PVDF layer coated Cu foil and MgF_2@PVDF layer. The deposition capacity of Li was 3.0 mAh cm^{-2}. The electrolyte used was 1.0 M LiTFSI in DOL/DME with 1.0 wt% $LiNO_3$.

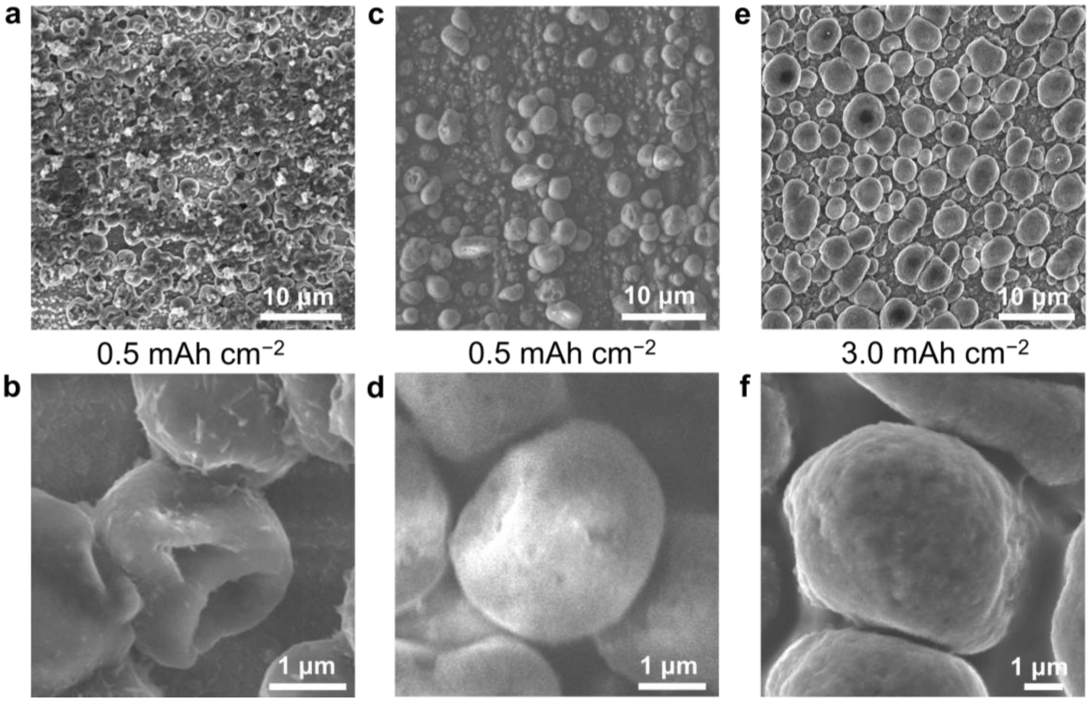

Supplementary Fig. 27 | Li deposits with and without an artificial passivation layer. **a, b** SEM images of Li deposits (0.5 mAh cm^{-2}) after soaking in the electrolyte without the protection of an artificial passivation layer. **c, d** SEM images of Li deposits (0.5 mAh cm^{-2}) after soaking in the electrolyte with the protection of an artificial passivation layer. **e, f** SEM images of Li deposits (3.0 mAh cm^{-2}) after soaking in the electrolyte with the protection of an artificial passivation layer. The electrolyte used was 1.0 M LiTFSI in DOL/DME with 1.0 wt% $LiNO_3$.

The Li deposits after soaking in the electrolyte suffered obvious corrosion with a large number of voids. In sharp contrast, the introduction of MgF_2@PVDF layer can prevent the corrosion of Li at both low and high deposition capacity of Li.

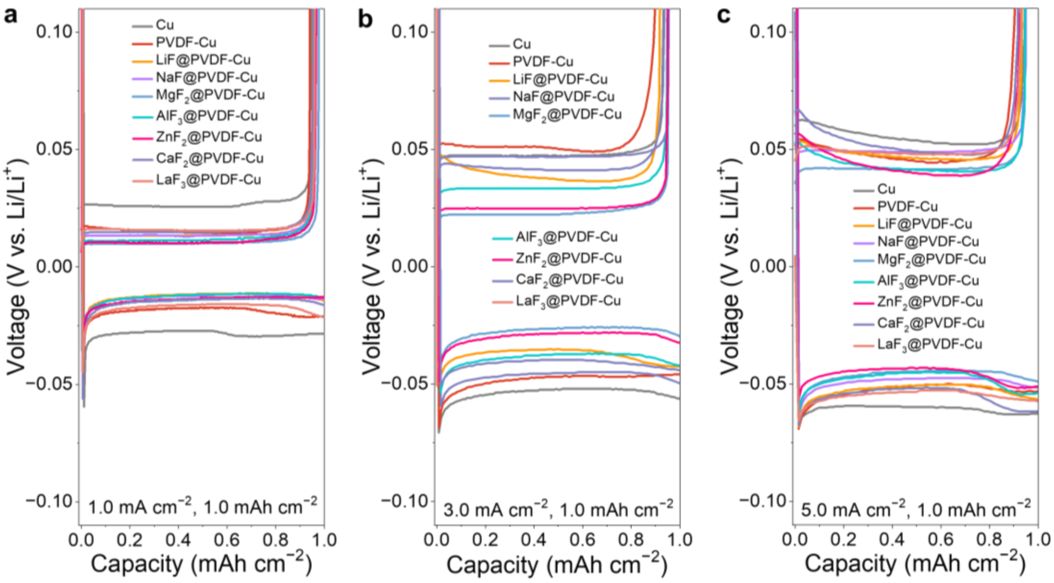

Supplementary Fig. 28 | Voltage profiles of various electrodes at various current densities. a 1.0 mA cm^{-2}. **b** 3.0 mA cm^{-2}. **c** 5.0 mA cm^{-2}. The electrolyte used was 1.0 M LiTFSI in DOL/DME with 1.0 wt% $LiNO_3$.

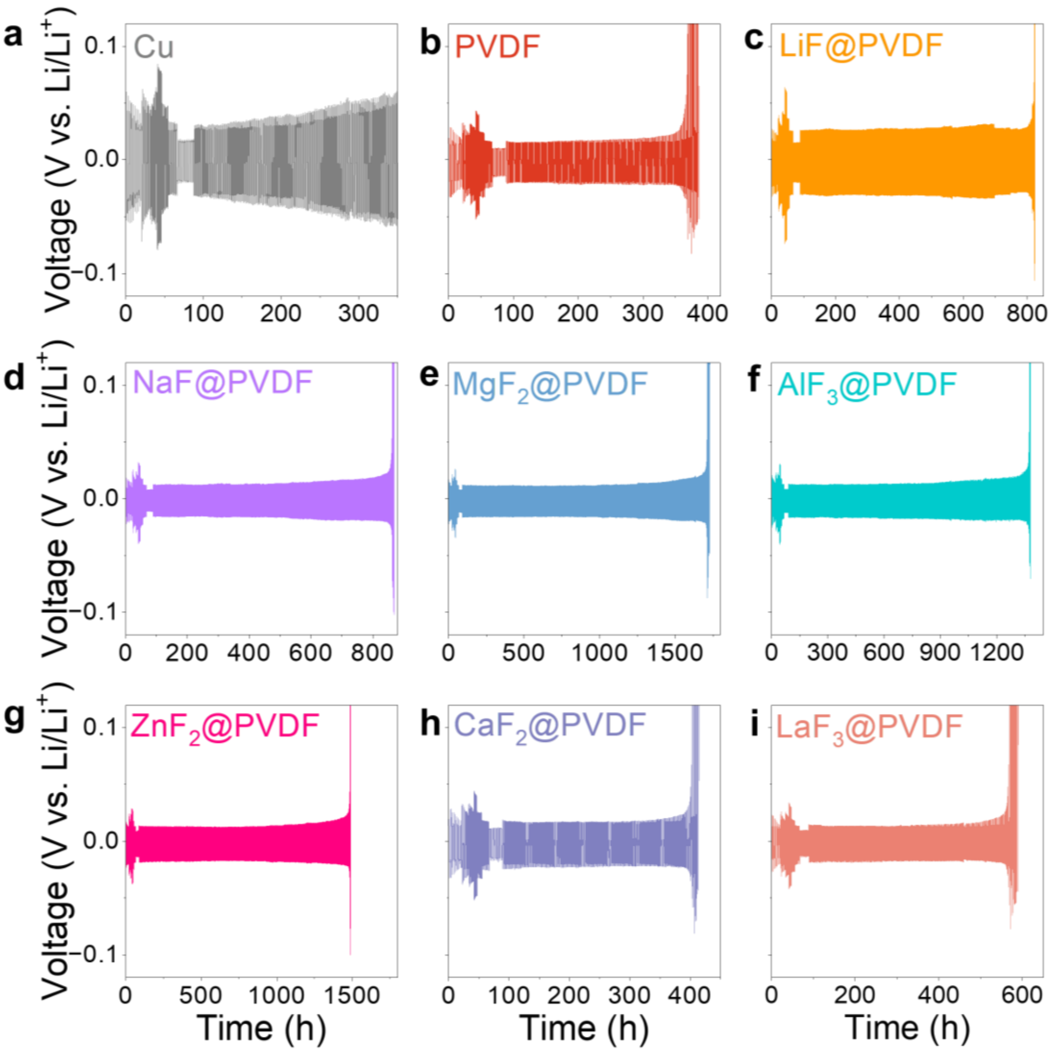

Supplementary Fig. 29 | Voltage profiles of symmetric cells at 1.0, 2.0, 3.0, 5.0, 3.0, 2.0, 1.0, and 2.0 mA cm^{-2}. a Bare Cu. **b** PVDF-Cu. **c** LiF@PVDF-Cu. **d** NaF@PVDF-Cu. **e** MgF_2@PVDF-Cu. **f** AlF_3@PVDF-Cu. **g** ZnF_2@PVDF-Cu. **h** CaF_2@PVDF-Cu. **i** LaF_3@PVDF-Cu. The electrolyte used was 1.0 M LiTFSI in DOL/DME with 1.0 wt% $LiNO_3$.

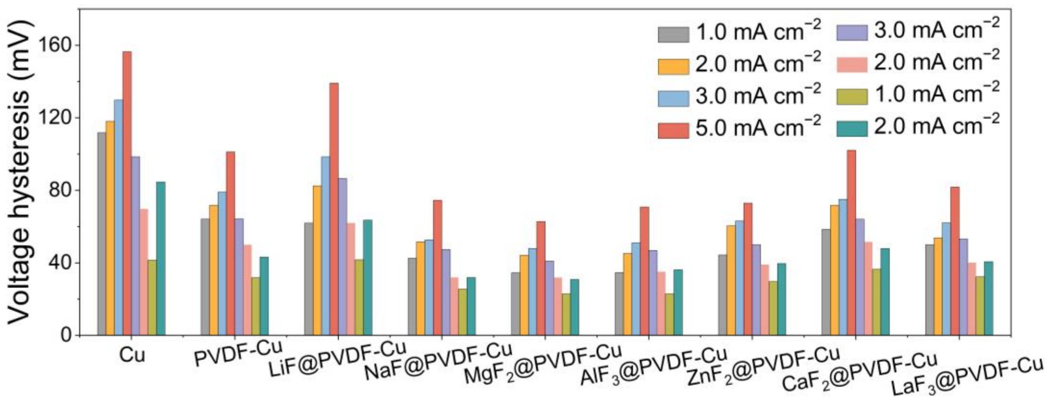

Supplementary Fig. 30 | Voltage hysteresis of symmetric cells with various electrodes at 1.0, 2.0, 3.0, 5.0, 3.0, 2.0, 1.0, and 2.0 mA cm^{-2}, respectively.

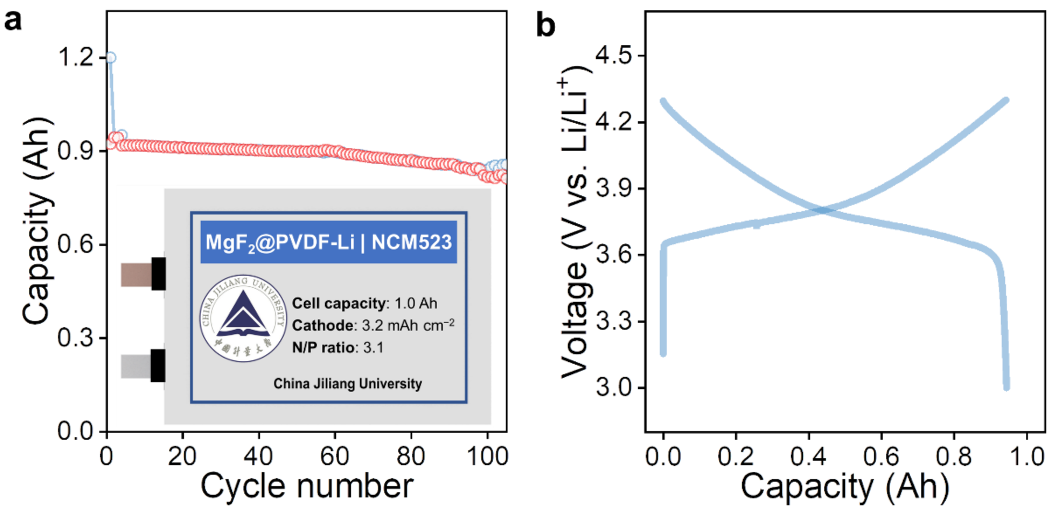

Supplementary Fig. 31 | Evaluation of Li||NCM523 pouch cell. a, b Cycling performance and the corresponding voltage profile of Li||NCM523 pouch cell. The cell was activated at 30 mA for three cycles, after which it was cycled with a charging/discharging current of 100 mA.

Supplementary tables

Supplementary Table 1. Techniques for exploring corrosion science.

Characterization techniques	Features	Refs.
**Microstructure and chemistry examination**		
Focused ion beam-scanning electron microscopy (FIB-SEM)	Visualizing the corrosion morphology on the surface and inside the materials	26
Cryo-electron transmission microscopy (cryo-TEM)	Atomically visualizing the nanostructure and chemistry of passive layer and corroded metals	27
High-angle annular dark-field scanning transmission electron microscopy (HAADF-STEM)	Detecting the atomic-resolution fine structures of corroded materials (*e.g.*, dislocations)	28
Electron backscatter diffraction (EBSD)	Measuring the grain orientation and size distribution of corroded materials	29
In-situ optical microscopy	Monitoring the corrosion behaviors and evolution process of metal surfaces	30
Atom probe tomography (APT)	Providing 3D compositional mapping of materials with sub-nanometer spatial resolution and no elemental mass limits	31
High-speed atomic force microscopy (HS-AFM)	Measuring stress corrosion cracking with high temporal and spatial resolution	32
**Electrochemical measurement**		
Scanning vibrating electrode technique (SVET)	Recording the local current density mappings and potentiodynamic gradients at metal/alloy surface	33
Kelvin probe force microscopy (KPFM)	Mapping the work function or surface potential of the sample with high spatial resolution	34
Localized electrochemical impedance spectroscopy (LEIS)	Measuring the local kinetics, capacitance, and resistance	35

Scanning electrochemical microscopy (SECM)	Monitoring the electron transfer with high spatial resolution, enabling the comprehension of corrosion mechanisms and mitigation strategies	36
Potentiodynamic polarization (PDP) method	Obtaining the corrosion potential to reveal the nobility and corrosion current of metals/alloys, indicating the corrosion rate	37
Zero resistance ammetry (ZRA) technique	Quantifying the galvanic coupling current between two dissimilar electrodes	38

Supplementary Table 2. The advances in researches on Li corrosion and protection.

Main content	Li protection strategy	Ref
The fast galvanic Li corrosion involving a Kirkendall-type mechanism	Passivation with a good electrical insulation and low electrolyte permeability	39
The galvanic corrosion of lithium-powder-based electrodes	Isolating copper from the electrolyte by additives, interfacial engineering, and physical vapor deposition techniques	40
The chemical corrosion of Li and continuous growth of the SEI during calendar ageing	Minimizing the surface area of Li and the rate of SEI growth, and reusing existing SEIs from previous cycles	41
The relationship between aging and cycling on self-discharge of Li metal batteries	Increasing the capacity of plated lithium, tuning electrolyte chemistry, designing rest protocols, and using lithiophilic coatings	42
A review on strategies to Li protection	Suppressing the dendrite growth and stabilizing interphase	43
A review on the artificial SEI for protecting Li	Reviewing the artificial SEI for protecting Li from corrosion by air and water	44
Chemically induced activity recovery of isolated lithium	A block copolymer coating to induce the reconnection of isolated Li	45
A review on reducing the Li corrosion by polysulfide	Guiding uniform Li plating/stripping, and reducing the concentration and reaction activity of polysulfide	46
The relation between chemical corrosion and Li depositing morphology	Controlling the stacking pressure during Li plating to reduce the porosity of Li deposits	47
The dynamic galvanic corrosion mechanism of Li metal during Li stripping process	A high-rate Li stripping protocol	25

The SEI dissolution during rest at open circuit has been quantified	Tuning the SEI composition and the physical and chemical properties of the electrolyte	48
The correlation between Li corrosion and dynamic SEI dissolution	A composite artificial passivation layer to inhibit the dissolution of native SEI and construct a superiorly stable interface	This work

Supplementary Table 3. Comparison between the literature on artificial SEI/protective layer and this work.

Material	Mechanism	Condition	CE	Ref
Interconnected hollow carbon nanospheres	Dendrite suppression and interfacial stabilization	1.0/1.0	~99% for 150 cycles	49
3D oxidized polyacrylonitrile nanofiber network	Guiding the Li^+ to form uniform Li deposits	3.0/1.0	97.4 for 120 cycles	50
Dihydroxyviolanthron layer	Stabilizing SEI and homogeniinge the deposition of Li metal	0.5/1.0	99.6 for 300 cycles	51
Lithiated Nafion film reinforced by organic filler	Producing a flexible interphase with uniform Li^+ diffusion	1.0/1.0	99.2% for ~150 cycles	52
Li_2S/Li_2Se protective layer	Constructing stable surface SEI with high ionic conductivity	1.0/1.0	98% for 360 cycles	53
Dual-protective interface of Prussian blue/rGO	Facilitating fast and uniform Li^+ flux to achieve uniform Li deposition	1.0/1.0	97.8% for 150 cycles	54
Poly(dimethylsiloxane) film with nanopores	Restraining Li dendrite growth and reducing the side reaction	1.0/1.0	93.2% for 100 cycles	55
A garnet LLZTO/lithiated Nafion layer	Affording uniform distribution/transport of Li^+, and accommodating Li deposition	1.0/1.0	97.7% for 150 cycles	56
A polymeric ionic liquid	Unifying Li^+ flux and	0.5/1.0	96.9%	57

	promoting a homogeneous Li plating		for 110 cycles	
Cu_3N/styrene butadiene rubber	Facilitating the transport of Li^+ and maintaining structural integrity	1.0/1.0	97.4% for 100 cycles	58
Supramolecular copolymer layer	Affording a high self-stabilization and strong adhesion	1.0/1.0	98.42% for 150 cycles	59
Poly(vinylidene-co-hexafluoropropylene)/LiF	Suppressing random Li deposition and the formation of isolated Li	1.0/1.0	96.3% for 60 cycles	60
Dynamic single-ion-conductive network	Mitigating side reactions and regulating Li deposition	1.0/1.0	94.9% for 250 cycles	61
Langmuir–Blodgett artificial SEIs	Inhibiting parasitic reactions and enabling uniform Li electrodeposition.	1.0/1.0	~96% for ~200 cycles	62
A reactive polymer composite derived SEI and 3D host	stabilizing the interface and preventing electrolyte decomposition	2.0/4.0	99.1% for 300 cycles	63
A salt-philic, solvent-phobic polymer coating	Promoting salt-derived SEI formation	0.5/1.0	99.5% for 10 cycles	64
A shielding layer of polymer and fluorides	Inhibiting SEI dissolution and Li corrosion	1.0/1.0	96.2% for 500 cycles	This work

Condition refers to the current density (mA cm^{-2}) and Li cycling capacity (mAh cm^{-2}), respectively.

Supplementary References

1 Zhang, X. *et al.* Uniform corrosion behavior of GZ51K alloy with long period stacking ordered structure for biomedical application. *Corros. Sci.* **88**, 1−5 (2014).

2 Frankel, G. S. Pitting corrosion of metals: A review of the critical factors. *J. Electrochem. Soc.* **145**, 2186 (1998).

3 Guo, X. *et al.* Self-accelerated corrosion of nuclear waste forms at material interfaces. *Nat. Mater.* **19**, 310−316 (2020).

4 Brau, F., Thouvenel-Romans, S., Steinbock, O., Cardoso, S. S. S. & Cartwright, J. H. E. Filiform corrosion as a pressure-driven delamination process. *Soft Matter* **15**, 803−812 (2019).

5 Clark, B., Clair, J. S. & Edwards, M. Copper deposition corrosion elevates lead release to potable water. *Journal AWWA* **107**, E627−E637 (2015).

6 Yan, C. *et al.* Evading strength-corrosion tradeoff in Mg alloys via dense ultrafine twins. *Nat. Commun.* **12**, 4616 (2021).

7 Michalske, T. A. & Freiman, S. W. A molecular interpretation of stress corrosion in silica *Nature* **295**, 511–512 (1982).

8 Olugbade, T. O., Omiyale, B. O. & Ojo, O. T. Corrosion, corrosion fatigue, and protection of magnesium alloys: Mechanisms, measurements, and mitigation. *J. Mater. eng. perform.* **31**, 1707−1727 (2021).

9 Song, J. & Curtin, W. A. Atomic mechanism and prediction of hydrogen embrittlement in iron. *Nat. Mater.* **12**, 145−151 (2013).

10 Wu, L., Ma, A., Zhang, L. & Zheng, Y. Intergranular erosion corrosion of pure copper tube in flowing NaCl solution. *Corros. Sci.* **201**, 110304 (2022).

11 Qin, Z. *et al.* Microstructure design to improve the corrosion and cavitation corrosion resistance of a nickel-aluminum bronze. *Corros. Sci.* **139**, 255−266 (2018).

12 Zahran, R. R. & Sedahmed, G. H. Mass-transfer-controlled impingement corrosion at the jet inlet zone of an annulus under turbulent flow. *Ind. Eng. Chem. Res.* **45**, 1160−1166 (2006).

13 Kaufman, J. *et al.* The effect of laser shock peening with and without protective coating on intergranular corrosion of sensitized AA5083. *Corros. Sci.* **194**, 109925 (2022).

14 Schroer, C., Wedemeyer, O., Novotny, J., Skrypnik, A. & Konys, J. Selective leaching of nickel and chromium from Type 316 austenitic steel in oxygen-containing lead–bismuth eutectic (LBE). *Corros. Sci.* **84**, 113−124 (2014).

15 Guo, X. *et al.* Understanding the fretting corrosion mechanism of zirconium alloy exposed to high temperature high pressure water. *Corros. Sci.* **202**, 110300 (2022).

16 Nimmervoll, M., Mori, G., Hönig, S. & Haubner, R. High-temperature corrosion of austenitic alloys in HCl and H_2S containing atmospheres under reducing conditions. *Corros. Sci.* **200**, 110214 (2022).

17 Videla, H. e. A. Prevention and control of biocorrosion. *Int. Biodeterior. Biodegrad.* **39**, 259–270 (2002).

18 Du, P. *et al.* Research progress towards the corrosion and protection of electrodes in energy-storage batteries. *Energy Storage Mater.* **57**, 371−399 (2023).

19 Shi, P. *et al.* Inhibiting intercrystalline reactions of anode with electrolytes for long-cycling lithium batteries. *Sci. Adv.* **8**, eabq3445 (2022).

20 Ryan, M. P., Williams, D. E., Chater, R. J., Hutton, B. M. & McPhail, D. S. Why stainless steel corrodes. *Nature* **415**, 770–774 (2002).

21 Zhao, Q. *et al.* On the crystallography and reversibility of lithium electrodeposits at ultrahigh capacity. *Nat. Commun.* **12**, 6034 (2021).

22 Bessone, J. B., Salinas, D. R., Mayer, C. E., Ebert, M. E. & Lorenz, W. J. An EIS study of aluminum barrier-type oxide films formed in different media. *Electrochim. Acta* **37**, 2283−2290 (1992).

23 Eswarappa Prameela, S. *et al.* Materials for extreme environments. *Nat. Rev. Mater.* **8**, 81−88 (2022).

24 Raja, V. S. Grand challenges in metal corrosion and protection research. *Front. Met. Alloy.* **1**, 894181 (2022).

25 Ding, J. F. *et al.* Dynamic galvanic corrosion of working lithium metal anode under practical conditions. *Adv. Energy Mater.*, **13**, 2204305 (2023).

26 Yang, Y. *et al.* One dimensional wormhole corrosion in metals. *Nat. Commun.* **14**, 988 (2023).

27 Lin, A. Y. W., Muller, A., Yu, X. X., Minor, A. M. & Marks, L. D. Early-stage NiCrMo oxidation revealed by cryo-transmission electron microscopy.

Ultramicroscopy **200**, 6−11 (2019).

28 Wei, X. X. *et al.* Enhanced corrosion resistance by engineering crystallography on metals. *Nat. Commun.* **13**, 726 (2022).

29 Wang, Y. M. *et al.* Additively manufactured hierarchical stainless steels with high strength and ductility. *Nat. Mater.* **17**, 63−71 (2018).

30 Zhang, S. *et al.* Concerning the stability of seawater electrolysis: a corrosion mechanism study of halide on Ni-based anode. *Nat. Commun.* **14**, 4822 (2023).

31 Luo, H. *et al.* A strong and ductile medium-entropy alloy resists hydrogen embrittlement and corrosion. *Nat. Commun.* **11**, 3081 (2020).

32 Moore, S. *et al.* Observation of stress corrosion cracking using real-time in situ high-speed atomic force microscopy and correlative techniques. *NPJ Mater. Degrad.* **5**, 3 (2021).

33 Bastos, A. C., Quevedo, M. C., Karavai, O. V. & Ferreira, M. G. S. Review—on the application of the scanning vibrating electrode technique (SVET) to corrosion research. *J. Electrochem. Soc* **164**, C973−C990 (2017).

34 Collins, L. *et al.* Probing charge screening dynamics and electrochemical processes at the solid-liquid interface with electrochemical force microscopy. *Nat. Commun.* **5**, 3871 (2014).

35 Jadhav, N. & Gelling, V. J. Review—the use of localized electrochemical techniques for corrosion studies. *J. Electrochem. Soc.* **166**, C3461−C3476 (2019).

36 Polcari, D., Dauphin-Ducharme, P. & Mauzeroll, J. Scanning electrochemical microscopy: A comprehensive review of experimental parameters from 1989 to 2015. *Chem. Rev.* **116**, 13234−13278 (2016).

37 Xu, W. *et al.* A high-specific-strength and corrosion-resistant magnesium alloy. *Nat. Mater.* **14**, 1229−1235 (2015).

38 Yang, L. & Yang, A. A. Communication—on zero-resistance ammeter and zero-voltage ammeter. *J. Electrochem. Soc.* **13**, C819−C821 (2017).

39 Lin, D. *et al.* Fast galvanic lithium corrosion involving a Kirkendall-type mechanism. *Nat. Chem.* **11**, 382−389 (2019).

40 Kolesnikov, A. *et al.* Galvanic corrosion of lithium-powder-based electrodes. *Adv. Energy Mater.* **10**, 2000017 (2020).

41 Boyle, D. T. *et al.* Corrosion of lithium metal anodes during calendar ageing

and its microscopic origins. *Nat. Energy* **6**, 487−494 (2021).

42 Merrill, L. C., Rosenberg, S. G., Jungjohann, K. L. & Harrison, K. L. Uncovering the relationship between aging and cycling on lithium metal battery self-discharge. *ACS Appl. Energy Mater.* **4**, 7589−7598 (2021).

43 Li, J. *et al.* Strategies to anode protection in lithium metal battery: A review. *InfoMat* **3**, 1333−1363 (2021).

44 Kang, D., Xiao, M. & Lemmon, J. P. Artificial solid-electrolyte interphase for lithium metal batteries. *Batteries & Supercaps* **4**, 445−455 (2020).

45 Ma, C. *et al.* Chemically induced activity recovery of isolated lithium in anode-free lithium metal batteries. *Nano Lett.* **22**, 9268−9274 (2022).

46 Bi, C.-X. *et al.* Protecting lithium metal anodes in lithium–sulfur batteries: A review. *Energy Mater. Adv.* **4**, 0010 (2023).

47 Lu, B. *et al.* Suppressing chemical corrosions of lithium metal anodes. *Adv. Energy Mater.* **12**, 2202012 (2022).

48 Sayavong, P. *et al.* Dissolution of the solid electrolyte interphase and its effects on lithium metal anode cyclability. *J. Am. Chem. Soc.* **145**, 12342–12350 (2023).

49 Zheng, G. *et al.* Interconnected hollow carbon nanospheres for stable lithium metal anodes. *Nat. Nanotechnol.* **9**, 618−623 (2014).

50 Liang, Z. *et al.* Polymer nanofiber-guided uniform lithium deposition for battery electrodes. *Nano Lett.* **15**, 2910−2916 (2015).

51 Chang, S. *et al.* In situ formation of polycyclic aromatic hydrocarbons as an artificial hybrid layer for lithium metal anodes. *Nano Lett.* **22**, 263−270 (2022).

52 Li, S. *et al.* A robust all-organic protective layer towards ultrahigh-rate and large-capacity Li metal anodes. *Nat. Nanotechnol.* **17**, 613−621 (2022).

53 Liu, F. *et al.* A mixed lithium-ion conductive Li_2S/Li_2Se protection layer for stable lithium metal anode. *Adv. Funct. Mater.* **30**, 2001607 (2020).

54 Fan, L. *et al.* A dual-protective artificial interface for stable lithium metal anodes. *Adv. Energy Mater.* **11**, 2102242 (2021).

55 Zhu, B. *et al.* Poly(dimethylsiloxane) thin film as a stable interfacial layer for high-performance lithium-metal battery anodes. *Adv. Mater.* **29**, 1603755 (2017).

56 Xu, R. *et al.* Dual-phase single-ion pathway interfaces for robust lithium metal in working batteries. *Adv. Mater.* **31**, 1808392 (2019).

57 Wu, J. *et al.* Polycationic polymer layer for air-stable and dendrite-free Li metal anodes in carbonate electrolytes. *Adv. Mater.* **33**, 2007428 (2021).

58 Liu, Y. *et al.* An artificial solid electrolyte interphase with high Li-ion conductivity, mechanical strength, and flexibility for stable lithium metal anodes. *Adv. Mater.* **29**, 1605531 (2017).

59 Wang, G. *et al.* Self-stabilized and strongly adhesive supramolecular polymer protective layer enables ultrahigh-rate and large-capacity lithium-metal anode. *Angew. Chem. Int. Ed.* **59**, 2055−2060 (2020).

60 Xu, R. *et al.* Artificial soft-rigid protective layer for dendrite-free lithium metal anode. *Adv. Funct. Mater.* **28**, 1705838 (2018).

61 Yu, Z. *et al.* A dynamic, electrolyte-blocking, and single-ion-conductive network for stable lithium-metal anodes. *Joule* **3**, 2761−2776 (2019).

62 Kim, M. S. *et al.* Langmuir–Blodgett artificial solid-electrolyte interphases for practical lithium metal batteries. *Nat. Energy* **3**, 889−898 (2018).

63 Gao, Y. *et al.* Polymer-inorganic solid-electrolyte interphase for stable lithium metal batteries under lean electrolyte conditions. *Nat. Mater.* **18**, 384−389 (2019).

64 Huang, Z. *et al.* A salt-philic, solvent-phobic interfacial coating design for lithium metal electrodes. *Nat. Energy* **8**, 577–585 (2023).
